# Identification of *Leptospira interrogans* Phospholipase C as a Novel Virulence Factor Responsible for Intracellular Free Calcium Ion Elevation during Macrophage Death

**DOI:** 10.1371/journal.pone.0075652

**Published:** 2013-10-04

**Authors:** Jing-Fang Zhao, Hong-Hu Chen, David M. Ojcius, Xin Zhao, Dexter Sun, Yu-Mei Ge, Lin-Li Zheng, Xu’ai Lin, Lan-Juan Li, Jie Yan

**Affiliations:** 1 State Key Laboratory for Diagnosis and Treatment of Infectious Diseases, The First Affiliated Hospital, Zhejiang University School of Medicine, Hangzhou, Zhejiang, P.R. China; 2 Department of Medical Microbiology and Parasitology, Zhejiang University School of Medicine, Hangzhou, Zhejiang, P.R. China; 3 Depatment of Clinical Laboratory, Zhejiang Provincial Hospital of Traditional Chinese Medicine, Hangzhou, Zhejiang, P.R. China; 4 Health Sciences Research Institute and Department Molecular Cell Biology, University of California Merced, Merced, California, United States of America; 5 Department of Neurology and Neuroscience, New York Presbyterian Hospital and Hospital for Special Surgery, Cornell University Weill Medical College, New York, New York, United States of America; Cornell University, United States of America

## Abstract

**Background:**

*Leptospira*-induced macrophage death has been confirmed to play a crucial role in pathogenesis of leptospirosis, a worldwide zoonotic infectious disease. Intracellular free Ca^2+^ concentration ([Ca^2+^]i) elevation induced by infection can cause cell death, but [Ca^2+^]i changes and high [Ca^2+^]i-induced death of macrophages due to infection of *Leptospira* have not been previously reported.

**Methodology/Principal Findings:**

We first used a Ca^2+^-specific fluorescence probe to confirm that the infection of *L. interrogans* strain Lai triggered a significant increase of [Ca^2+^]i in mouse J774A.1 or human THP-1 macrophages. Laser confocal microscopic examination showed that the [Ca^2+^]i elevation was caused by both extracellular Ca^2+^ influx through the purinergic receptor, P_2_X_7_, and Ca^2+^ release from the endoplasmic reticulum, as seen by suppression of [Ca^2+^]i elevation when receptor-gated calcium channels were blocked or P_2_X_7_ was depleted. The LB361 gene product of the spirochete exhibited phosphatidylinositol phospholipase C (L-PI-PLC) activity to hydrolyze phosphatidylinositol-4,5-bisphosphate (PIP_2_) into inositol-1,4,5-trisphosphate (IP_3_), which in turn induces intracellular Ca^2+^ release from endoplasmic reticulum, with the Km of 199 µM and Kcat of 8.566E-5 S^-1^. Secretion of L-PI-PLC from the spirochete into supernatants of leptospire-macrophage co-cultures and cytosol of infected macrophages was also observed by Western Blot assay. Lower [Ca^2+^]i elevation was induced by infection with a LB361-deficient leptospiral mutant, whereas transfection of the LB361 gene caused a mild increase in [Ca^2+^]i. Moreover, PI-PLCs (PI-PLC-β3 and PI-PLC-γ1) of the two macrophages were activated by phosphorylation during infection. Flow cytometric detection demonstrated that high [Ca^2+^]i increases induced apoptosis and necrosis of macrophages, while mild [Ca^2+^]i elevation only caused apoptosis.

**Conclusions/Significance:**

This study demonstrated that *L. interrogans* infection induced [Ca^2+^]i elevation through extracellular Ca^2+^ influx and intracellular Ca^2+^ release cause macrophage apoptosis and necrosis, and the LB361 gene product was shown to be a novel PI-PLC of *L. interrogans* responsible for the [Ca^2+^]i elevation.

## Introduction

Leptospirosis caused by pathogenic *Leptospira* species is a world-spread zoonotic infectious disease [1]. The disease has been prevalent in most countries in Southeast Asia and South America [2], [3]. However, in recent years, human leptospirosis cases have also been frequently reported in North America and Europe [4]–[6], and the disease was identified as an emerging global public health problem [1], [7].

Many animals serve as the natural hosts of pathogenic *Leptospira* species [8]. When individuals come in contact with soil or water contaminated with leptospire-containing urine from infected animals, the leptospires invade into human body through the skin or mucosa to cause leptospirosis [9], [10]. The mild cases of infection exhibit influenza-like manifestations such as fever and myalgia, while the severe cases frequently result in death due to respiratory failure caused by pulmonary diffuse hemorrhaging and meningitis or renal failure due to renal injury and jaundice [3], [4], [9], [10]. However, until now, the pathogenic mechanisms of *Leptospira* infection remain poorly understood.

Macrophages and neutrophils play an important role in innate immunity against infection through phagocytosis of microbial pathogens including *Leptospira*. However, unlike many other bacterial pathogens, only macrophages can kill the phagocytosed leptospires in individuals who do not have specific antibodies against *Leptospira* [9], [11], [12]. Therefore, macrophages act as a crucial phagocyte in the host defense mechanisms against leptospiral infection in unvaccinated individuals, and ability to evade phagocytosis by macrophages contributes to virulence of pathogenic *Leptospira* species [13]–[15].

Infection results from interaction between microbial pathogens and hosts [16], [17]. In order for the hosts to respond to pathogen, or for the pathogen to resist the innate immune response of the host, both the pathogens and hosts must modify significantly their metabolism and gene expression profiles [18], [19]. For instance, intracellular free calcium ion (Ca^2+^), an important intracellular messenger with multiple physiological functions, is increased when cells are infected with some bacterial pathogens [20]. Thus, *Helicobacter pylori* or *Campylobacter jejuni* cause an elevation of intracellular free Ca^2+^ concentration ([Ca^2+^]i) through Ca^2+^ release and/or influx mechanisms in gastric mucous epithelial cells or in intestinal epithelial cells during infection [21], [22]. The high [Ca^2+^]i in macrophages caused by infection with *Listeria monocytogenes* or *Brucella abortus* are involved in bacterial invasion and escape from phagocytotic vesicles for intracellular replication [23], [24]. In particular, high [Ca^2+^]i can induce cell death, in which a mild [Ca^2+^]i increases typically stimulates cell apoptosis while a high [Ca^2+^]i change results in cell necrosis [25]. Our previous studies confirmed that *L. interrogans*, a predominant pathogenic *Leptospira* species, could be phagocytized by human or mouse macrophages, but infection induces macrophage apoptosis and necrosis [14]–[16]. However, the change of [Ca^2+^]i in *L. interrogans*-infected macrophages and the role of high [Ca^2+^]i in *Leptospira*-induced macrophage death had not been investigated yet.

Phospholipase (PL) is a group of enzymes in eukaryotes and prokaryotes that can be classified into the PLA, PLC, PLD and sphingonyelinase (SMsae) subfamilies according to the specificity of their substrates and diversity of products [26]. PLC is also divided into PC-PLC, which hydrolyzes phosphatidyl choline (PC) to produce phosphorylcholine and diacylglycerol (DAG), and PI-PLC, which hydrolyzes phosphatidylinositol-4,5-bisphosphate (PIP_2_) to produce inositol-1,4,5-trisphosphate (IP_3_) and DAG. IP_3_ can bind to the IP_3_-receptor (IP_3_R) on endoplasmic reticulum to trigger release of Ca^2+^ from the endoplasmic reticulum into cytosol, resulting in[Ca^2+^]i elevation [27].

Until now, except for a PI-PLC produced by *L. monocytogenes*, few bacterial PLC have been well characterized [28]. Our bioinformatic analysis indicated that there are three PLC domain-containing genes (LA0543, LA2250 and LB361) in chromosomal DNA of *L. interrogans* serogroup Icterohaemorrhagiae serovar Lai strain Lai [29]. However, the PLC activity of the three gene products and the possible role of *Leptospira*-induced [Ca^2+^]i elevation in macrophage death have not been determined yet.

In the present study, we investigated the changes of [Ca^2+^]i in macrophages during infection with *L. interrogans* strain Lai, the dependence of [Ca^2+^]i on Ca^2+^ influx through membrane calcium channels or release from endoplasmic reticulum, and the correlation between [Ca^2+^]i changes and macrophage apoptosis and necrosis. Subsequently, we characterized biochemically the PI-PLC or PC-PLC enzymatic activity of the proteins expressed by the three leptospiral PLC-domain-containing genes and demonstrated the function of leptospiral PI-PLC in [Ca^2+^]i elevation and macrophage death during infection. The results of this study identify a novel PI-PLC of *L. interrogans* and show its the role in the [Ca^2+^]i elevation and death of infected macrophages.

## Materials and Methods

### Leptospiral Strains and Culture

Seven pathogenic *L. interrogans* strains and two non-pathogenic *L. biflexa* strains belonging to different serogroups and serovars (see Supplemental Materials) were cultivated in Ellinghausen-McCullough-Johnson-Harris (EMJH) liquid medium at 28°C [30].

### Cell Lines and Culture

A mouse macrophage line (J774A.1) and a human monocytic line (THP-1) were provided by the Cell Bank of the Institute of Cytobiology, Chinese Academy of Science. The cells were maintained in RPMI 1640 medium (Gibco, USA), supplemented with 10% fetal calf serum (FCS, Gibco), 100 U ml^−1^ penicillin and 100 µg ml^−1^ streptomycin (Sigma, USA) at 37°C in an atmosphere of 5% CO_2_. In particular, THP-1 cells were pre-treated with 10 ng ml^−1^ phorbol 12-myristate 13-acetate (PMA, Sigma) at 37°C for 48 h to differentiate them into macrophages before use [31].

### Bioinformatic Analysis of Putative Leptospiral PLC-encoding genes

PLC-domains in the LA0543, LA2250 and LB361 genes of *L. interrogans* serogroup Icterohaemorrhagiae serovar Lai strain Lai (GenBank accession No.: NC_004342) were predicted by using SWISS-MODEL and InterProScan softwares [32], [33]. Signal peptides and transmembrane regions in sequences of the three genes were analyzed using SignalP 3.0 and TMHMM 2.0 softwares.

### Detection of Target Genes in Different Leptospiral Strains

The distribution of LA0543, LA2250 and LB361 genes in seven pathogenic *L. interrogans* strains and two non-pathogenic *L. biflexa* strains belonging to different serogroups and serovars were detected by PCR and sequencing. The experimental details are given in Materials S1.

### Expression and Extraction of Target Recombinant Proteins

The signal peptide sequence-lacking LA0543 and LA2250 genes and the entire LB361 gene of *L. interrogans* strain Lai were amplified by PCR. After sequencing, the LA0543, LA2250 and LB361 gene segments were linked with the linearized pET42a plasmid (Novagen, USA) to form recombinant expression vectors, respectively, and then transformed into *E. coli* BL21DE3 (Novagen) for expression. The expressed soluble recombinant proteins were examined by SDS-PAGE and then extracted by Ni-NTA affinity chromatography (Figure S2B). The details for the expression and extraction of recombinant proteins expressed by the three leptospiral genes are given in Materials S1.

### Preparation of Antisera and IgGs

Details about the preparation of antisera and IgGs against the recombinant proteins expressed by the LA0543, LA2250 and LB361 genes of *L. inetrrogans* strain Lai are given in Materials S1.

### Detection of PLC Activity of Leptospiral Recombinant Proteins

The PC-PLC or PI-PLC activity of recombinant proteins expressed by the LA0543, LA2250 and LB361 genes of *L. interrogans* strain Lai was detected by the ρ-nitrophenylphosphorylcholine (NPPC) assay or IP_3_ fluorescence polarization assay as previously reported [34], [35]. Briefly, in the NPPC assay, 90 µl borax-HCl buffer (100 mM, pH 7.5) containing 0.1, 1 or 10 µg each of the recombinant proteins was mixed with 10 µl borax-HCl buffer containing 100 mM NPPC (Sigma) for a 30-min incubation at 37°C. The ρ-nitrophenol released due to NPPC hydrolysis was quantified using a spectrophotometer (Bio-Rad, USA) at OD_410_, and the PC-PLC activity was calculated based on the standard curve created with different concentrations of ρ-nitrophenol (Sigma). In the IP_3_ fluorescence polarization assay, 50 µl reaction buffer (100 mM KCl, 1.9 mM CaC1_2_, 2 mM EGTA, 0.5 mg ml^−1^ bovine serum albumin and 0.1% sodium deoxycholate in 50 mM HEPES buffer, pH 7.0) containing 0.1, 1 or 10 µg each of the recombinant proteins was added with 400 µM PIP_2_ substrate (Echelon, USA). After incubation at 37°C for 10 min, the mixture was added with 250 µl chloroform-methanol-HCl (500∶500∶3, V:V:V) for a short vortex, and then added with 100 µl 5 mM EGTA-1 M HCl solution to terminate the reaction. The mixture was centrifuged at 500×g for 10 min and the aqueous phase was harvested to detect the IP_3_ concentration using a HitHunter™ IP_3_ Assay Kit (DiscoveRx Corp, USA) and a SpectraMax M5 Reader (Molecular Devices, USA) with fluorescence polarization spectrograph at FP-model according to the manufacturer’s protocol. The PI-PLC activity was calculated based on the standard curves created with different concentrations of IP_3_ (DiscoveRx Corp). In these assays, bovine serum albumin (BSA, Sigma) was used as the control, while 10 µM U73122, a mammalian cell PI-PLC blocker [36], was used to inhibit the PI-PLC activity of recombinant proteins.

### Determination of Km and Kcat Values of rL-PI-PLC

To determine the enzyme kinetic parameters (Km and Kcat) of recombinant protein expressed by the LB361 gene of *L. interrogans* strain Lai (rL-PI-PLC), 60, 80, 100, 200, 400, 600 or 800 µM of PIP_2_ substrate (Echelon) in 50 µl of the reaction buffer, as described above, was mixed with 1 µg rL-PI-PLC for a 10-min incubation at 37°C. IP_3_ concentrations in the mixtures due to PI-PLC-based hydrolysis of PIP_2_ substrate were detected by the IP_3_ fluorescence polarization assay, as described above. The Km and Kcat values of rL-PI-PLC were calculated using the double reciprocal Lineweaver-Burk plot according to the standard curves created with different concentrations of IP_3_ (DiscoveRx Corp) [37].

### Identification of Membrane Calcium Channels in Macrophages

J774A.1 or THP-1 cells were collected by a 500×g centrifugation for 15 min at 4°C. After washing with PBS and centrifugation, total membrane proteins of the cells were extracted using a Membrane Protein Extraction Kit (AbCam, USA). The protein concentration in the membrane protein samples was quantified using a BCA Protein Quantitative Kit (Beyotime Biotech, China). Using 1∶500 diluted rabbit anti-voltage-gated Cav3.1, Cav3.2, Cav3.3 or Cav2.3 calcium channel protein-IgG (AbCam), or anti-receptor-gated P_2_X_1_, P_2_X_2_, P_2_X_3_, P_2_X_4_, P_2_X_5_, P_2_X_6_ or P_2_X_7_ calcium channel protein-IgG (Santa Cruz, USA) as the primary antibody, and 1∶3000 diluted HRP-conjugated goat anti-rabbit-IgG (Jackson ImmunoResearch, USA) as the secondary antibody, Western Blot assays were performed to detect the membrane calcium channels of J774A.1 or THP-1 cells.

### Detection of Leptospires in Macrophages during Infection

J774A.1 or THP-1 cells (10^6^ cells per well) were seeded in 6-well culture plates (Corning, USA) for a 12-h incubation at 37°C to form monolayers. Freshly cultured *L. interrogans* strain Lai in EMJH medium was precipitated by a 17200×g centrifugation at 15°C for 15 min. After washing twice with PBS, the leptospiral pellet was suspended in antibiotic-free 2.5% FCS RPMI-1640 medium for counting leptospires with a Petroff-Hausser counting chamber under a dark-field microscope (Fisher Scientific, USA) [38]. The cell monolayers were infected with the spirochete at a multiplicity of infection (MOI) of 100 (100 leptospires per cell) for 0.5 or 1 h [15]. The leptospires in macrophages were detected by transmission electron microscopy and laser confocal microscopy as previously described [19].

### Measurement of Target Leptospiral Gene-mRNAs during Infection

J774A.1 or THP-1 cell monolayers (10^6^ cells per well) were infected with *L. interrogans* strain Lai (10^8^) at a MOI of 100 for 0.5, 1, 2, 4 or 8 h. The cultures were treated with 0.05% NaTDC-PBS to lyse cells [19], followed by a 17,200×g centrifugation at 4°C for 15 min to precipitate leptospires. Total RNAs in the leptospires were extracted using Trizol reagent (Invitrogen, China) plus digestion with RNase-free DNase (TaKaRa, China). cDNAs from the RNAs were synthesized by reverse transcription (RT) using a PrimeScript® RT reagent Kit (TaKaRa). Using each of the cDNAs as the template, mRNA levels of the LA0543, LA2250 or LB361 gene in the leptospires were assessed by real-time quantitative PCR (qPCR) using a SYBR® Premix Ex-Taq™ II Kit (TaKaRa) in an ABI 7500 Real-Time PCR System (ABI, USA). The primers used in the RT-qPCR are shown in Table S1. In the RT-qPCRs, the leptospiral 16S rRNA gene was used as the internal reference [39]. The RT-qPCR data were analyzed using the ΔΔCt model and randomization test in REST 2005 software [40].

### Determination of Leptospiral Protein Secretion during Infection

J774A.1 or THP-1 cell monolayers (10^6^ cells per well) were infected with *L. interrogans* strain Lai (10^8^) at a MOI of 100 for 0.5, 1, 2 or 4 h as above. The supernatants of cultures were collected for a 15-min centrifugation at 17,200×g (4°C) to remove extracellular leptospires. After washing thoroughly with PBS, the cell monolayers were lysed with 0.05% NaTDC-PBS [19]. The lysates were centrifuged as above to remove intracellular leptospires, and the cytosol specimens were harvested. In addition, the supernatant from culture of the spirochete (10^8^) in EMJH medium was also collected by centrifugation as above. Total proteins in all the supernatant and cytosol samples were extracted by trichloroacetic acid precipitation as previously described [41]. Using 1∶200 diluted rabbit-IgG against the recombinant protein of the LA0543, LA2250 or LB361 gene as the primary antibody, and 1∶3000 diluted HRP-conjugated goat anti-rabbit IgG (Jackson ImmunoResearch) as the secondary antibody, Western Blot assays were performed to detect the proteins expressed by the LA0543, LA2250 and LB361 genes. In these assays, a leptospiral nonsecreted cytoplasmic protein, HlyX, was used as the control, and the recombinant HlyX protein (rHlyX) and rHlyX-IgG were provided by our laboratory [41].

### Generation and Identification of the LB361 Gene-deleted or Complemented Mutant

Plasmid pUC19 was used for LB361 gene deletion (ΔLB361) and complementation (CΔLB361) since only the LB361 gene product was confirmed to have PI-PLC activity. Briefly, a suicide plasmid pUC19^5’arm-kan-3′arm^ was constructed and then electrotransformed into wild-type *L. interrogans* strain Lai to replace the LB361 gene with the kanamycin-resistant cassette sequence (kan) through allelic exchange by the 5′ and 3′ homologous arm sequences from the upstream and downstream regions of the gene to generate a ΔLB361 mutant. Conversely, a recombinant plasmid pUC19^5’arm-LB361-spc-3′arm^ was constructed and then electrotransformed into the ΔLB361 mutant to replace the kan gene with the LB361-spc segment through allelic exchange by the 5′ and 3′ homologous arm sequences to generate a CΔLB361 mutant. The deletion in the ΔLB361 mutant and the complementation in the CΔLB361 mutant were confirmed by PCR, sequencing, and Western Blot assay (Figure S3 and S4A). The details for the generation and identification of the ΔLB361 and CΔLB361 mutants are given in Materials S1.

### Generation and Identification of LB361 or *chpI* Gene-transfected Macrophages

pCMV-Tag2C, a prokaryote-eukaryote shuttle plasmid, is often used to transfect prokaryotic genes into different mammalian cells. Briefly, a recombinant pCMV-Tag2C containing the LB361 gene of *L. interrogans* strain Lai (pCMV-Tag2C^LB361^) was constructed and then transfected into J774A.1 or THP-1 cells using a Lipofectamine 2000 Transfection Kit (Invitrogen) or a Human Monocyte Nucleofector Kit (Lonza, Germany). The expression of the LB361 gene product in the LB361 gene-transfected macrophages was confirmed by Western Blot and immunofluorescence assays (Figure S4B and E). In addition, the *chpI* gene of *L. interrogans* strain Lai was transfected into J774A.1 or THP-1 cells, and ChpI protein expression in the transfected macrophages was determined by Western Blot assay (Figure S4C). Our recent study confirmed that the *chpI* gene product has no cytotoxicity to macrophages (the manuscript has been submitted to the journal of BMC Microbiology), and the *chpI* gene-transfected macrophages were used as the negative controls to determine the function of the LB361 gene product in host macrophages. The details about the generation and identification of LB361 or *chpI* gene-transfected J774A.1 and THP-1 cells are given in Materials S1.

### Generation and Identification of P_2_X_7_-depleted Macrophages

To determine the role of the P_2_X_7_ calcium channel in extracellular Ca^2+^ influx, the P_2_X_7_ gene in J774A.1 or THP-1 cells was depleted with siRNA interference. The absence of P_2_X_7_ protein in the P_2_X_7_-depleted J774A.1 or THP-1 cells was confirmed by Western Blot assay (Figure S4D). The details about the generation of P_2_X_7_-depleted J774A.1 and THP-1 cells with siRNA interference and their characterization are given in Materials S1.

### Detection of PI-PLC Phosphorylation of J774A.1 and THP-1 Cells during Infection

J774A.1 or THP-1 cell monolayers (10^6^ cells per well) were infected with *L. interrogans* strain Lai (10^8^) at a MOI of 100 for 0.5, 1, 2 or 4 h. After trypsinization, washing with PBS and centrifugation, the precipitated cells were lysed with 0.05% NaTDC-PBS [19], followed by a 17,200×g centrifugation at 4°C for 15 min to harvest leptospire-free supernatants. Using 1∶500 diluted rabbit-IgG against total PLC-β3 or PLC-γ1, pSer537 in PLC-β3, or pTyr759, pTyr783 or pTyr1217 in PLC-γ1 (Santa Cruz) as the primary antibody, and 1∶3000 diluted HRP-conjugated goat anti-rabbit-IgG (Jackson ImmunoResearch) as the secondary antibody, Western Blot assays were performed to detect the phosphorylation of PLC-β3 and PLC-γ1 in the supernatant samples. In this assay, β-actin was used as the control.

### Measurement of [Ca^2+^]i in Macrophages during Infection

J774A.1 or THP-1 cells (10^5^ cells per well) were seeded in 12-well culture plates (Corning) for a 12-h incubation at 37°C. The cell monolayers were washed thoroughly with D-Hank’s buffer and then incubated in 100 µl of 0.2% BSA RPMI-1640 medium containing 10 µM fluorescent calcium indicator fluo-4/AM (Molecular Probes, USA) at 37°C for 1 h, followed by a 30-min incubation with 2.5% FCS RPMI-1640 medium for AM de-esterification to release the indicator. After washing with D-Hank’s buffer again, the cell monolayers were infected with *L. interrogans* strain Lai (10^7^) for a 120-min contiguous detection at 37°C in a laser confocal microscope (type LSM510, Zeiss, Germany) to measure the fluorescence signal intensity reflecting intracellular free Ca^2+^ concentration ([Ca^2+^]i) (494 nm excitation wavelength and 516 nm emission wavelength) according to the manufacturer’s protocol. In the detection procedure, the images of leptospire-cell co-cultures incubated for 15, 30, 45, 60, 90 and 120 min were analyzed. The change of [Ca^2+^]i in the leptospire-infected J774A.1 or THP-1 cells was calculated as the following formula: the fluorescence intensity in 500 cells infected with the spirochete for different times (Fx)/the fluorescence intensity in the same number of cells before infection (F_0_) ×100% [36], [42]. In this protocol, the same number of normal J774A.1 or THP-1 cells without infection, and J774A.1 or THP-1 cells infected with the same number of spirochete that had been killed by heating at 100°C for 5 min [8], were used as the controls.

### Determination of Intracellular free Ca^2+^ Source in Macrophages during Infection

EGTA is an extracellular Ca^2+^ chelator, while BAPTA/AM is an intracellular free Ca^2+^ chelator [43.44]. Neomycin is a blocker of IP_3_ production [45]. SKF96365 is a receptor-gated Ca^2+^ channel blocker [46], while verapamil or mibefradil acts as blocker of L-type or T-type voltage-gated Ca^2+^ channels [47], [48]. To determine the source of intracellular free Ca^2+^ in J774A.1 or THP-1 cells (10^5^ per well) during infection with *L. interrogans* strain Lai (10^7^), the two macrophage monolayers were pre-treated with 2 mM EGTA (Sigma), 100 µM BAPTA/AM (Sigma), 1.2 mM neomycin (Sigma), 20 µM SKF96365 (Sigma), 100 µM verapamil (Sigma) or 10 µM mibefradil (Sigma) for 30 min at 37°C [43]–[48]. The subsequent experimental steps for [Ca^2+^]i detection were the same as described above. In this assay, the normal J774A.1 or THP-1 cells before infection, the chelator or blocker-untreated, and P_2_X_7_-depleted J774A.1 or THP-1 cells infected with the spirochete were used as the controls.

### Determination of the Role of Cellular or Leptospiral PI-PLC in [Ca^2+^]i Elevation of Macrophages

J774A.1 or THP-1 cell monolayers (10^5^ cells per well) were infected with the ΔLB361 or CΔLB361 mutant or wild-type *L. interrogans* strain Lai (10^7^) for a 120-min contiguous incubation at 37°C. To further determine the roles of cellular PI-PLC or L-PI-PLC in [Ca^2+^]i elevation of macrophages during infection with the spirochete, J774A.1 or THP-1 cell monolayers were pre-treated with 2 mM extracellular Ca^2+^ chelator EGTA (Sigma) [43], 10 µM mammalian cell PI-PLC blocker U73122 (Sigma) [36] and/or 1.2 mM IP_3_ production blocker neomycin (Sigma) [45], and then infected with the ΔLB361 or CΔLB361 mutant or wild-type strain (10^7^). The subsequent experimental steps for [Ca^2+^]i detection were the same as described above. In this detection, the normal J774A.1 or THP-1 cells before infection were used as the controls.

### Detection of Macrophage Death by Flow Cytometry

J774A.1 or THP-1 cell monolayers (10^5^ cells per well) were infected with the ΔLB361 or CΔLB361 mutant or wild-type *L. interrogans* strain Lai (10^7^) for 0.5, 1 or 2 h. After trypsinization, washing with PBS and centrifugation, the harvested cell pellets were suspended in annexin-binding buffer. The cell suspensions were treated with both Alexa Fluor® 488-annexin V and propidium iodide (PI) dyes in a Vybrant® Apoptosis Assay Kit (Invitrogen) for 15 min at room temperature. The stained cells were detected using a flow cytometer (FC500 MCL, Beckman, USA) to distinguish the cells in early apoptotic cells (annexin V^+^/PĪ) from post-apoptosis/necrosis (annexin V^+^/PI^+^). In addition, the J774A.1 or THP-1 cells (10^5^ per well), which were pretreated with 100 µM intracellular free Ca^2+^ chelator BAPTA/AM, 2 mM extracellular Ca^2+^ chelator EGTA, 2 mM EGTA plus 10 µM mammalian cell PI-PLC inhibitor U73122 or 1.2 mM IP_3_ production blocker neomycin [36], [43]–[45], were infected with the ΔLB361LB mutant or wild-type strain (10^7^) for the indicated times. Cell death was detected by flow cytometry as above. In the detection, the normal J774A.1 or THP-1 cells before infection as well as P_2_X_7_-depleted J774A.1 or THP-1 cells before or after infection with the spirochete were used as the controls.

### Enumeration of Leptospiral Colony-forming Units from Macrophages

J774A.1 or THP-1 cell monolayers (10^4^ cells per well) were infected with the ΔLB361 or CΔLB361 mutant or wild-type *L. interrogans* strain Lai (10^6^) for 0.5, 1 or 2 h. After removal of supernatants and trypsinization, the J774A.1 and THP-1 cells were collected by a 500×g centrifugation for 15 min at 4°C, followed by lysis with 0.05% NaTDC-PBS [19]. The lysates were centrifuged at 17,200×g for 15 min (4°C) to precipitate intracellular leptospires. Serial dilutions of the leptospiral pellets were inoculated onto EMJH-agar plates and then incubated at 28°C for three weeks [16]. The leptospiral colony-forming units (CFUs) on plates were enumerated after incubation.

### Detection of IP_3_ Levels in LB361 Gene-transfected Macrophages

The LB361 gene-transfected J774A.1 or THP-1 cells were incubated in 10% FCS RPMI-1640 at 37°C for 0.5, 1 or 2 h. After trypsinization, washing with PBS and centrifugation, the cell pellets were lysed with 0.05% NaTDC-PBS and then centrifuged at 17,200×g for 15 min (4°C) to remove leptospires [19]. The cytosol specimens were harvested to measure the intracellular IP_3_ levels by IP_3_ fluorescence polarization spectrography as described above. In the detection, the normal J774A.1 and THP-1 cells without transfection, and wild-type pCMV-Tag2C or *chpI* gene-transfected J774A.1 or THP-1 cells were used as the controls.

### Detection of [Ca^2+^]i and Apoptosis of in LB361 Gene-transfected Macrophages

To avoid the influence of extracellular Ca^2+^ influx on [Ca^2+^]i, J774A.1 and THP-1 cells were pretreated with 2 mM extracellular Ca^2+^ chelator EGTA (Sigma) at 37°C for 30 min [43]. The [Ca^2+^]i or apoptosis of the macrophages at different times was detected by laser confocal microscopy or flow cytometry as described above. In the detections, the normal J774A.1 or THP-1 cells without transfection, and wild-type pCMV-Tag2C-transfected or *chpI*-gene-transfected J774A.1 or THP-1 cells were used as the negative controls. In addition, the pCMV-Tag2C^LB361^-transfected J774A.1 or THP-1 cells pretreated with 1.2 mM IP_3_ production blocker neomycin were also used as the controls to further determine the function of the LB361 gene product.

### Data Analysis

Data from a minimum of three experiments were averaged and presented as mean ± standard deviation (SD). One-way analysis of variance (ANOVA) followed by Dunnett’s multiple comparisons test were used to determine significant differences. Statistical significance was defined as *p*<0.05.

## Results

### Distribution of PLC-domain-containing LA0543, LA2250 and LB361 Genes in Different Leptospiral Strains

**Figure 1:**
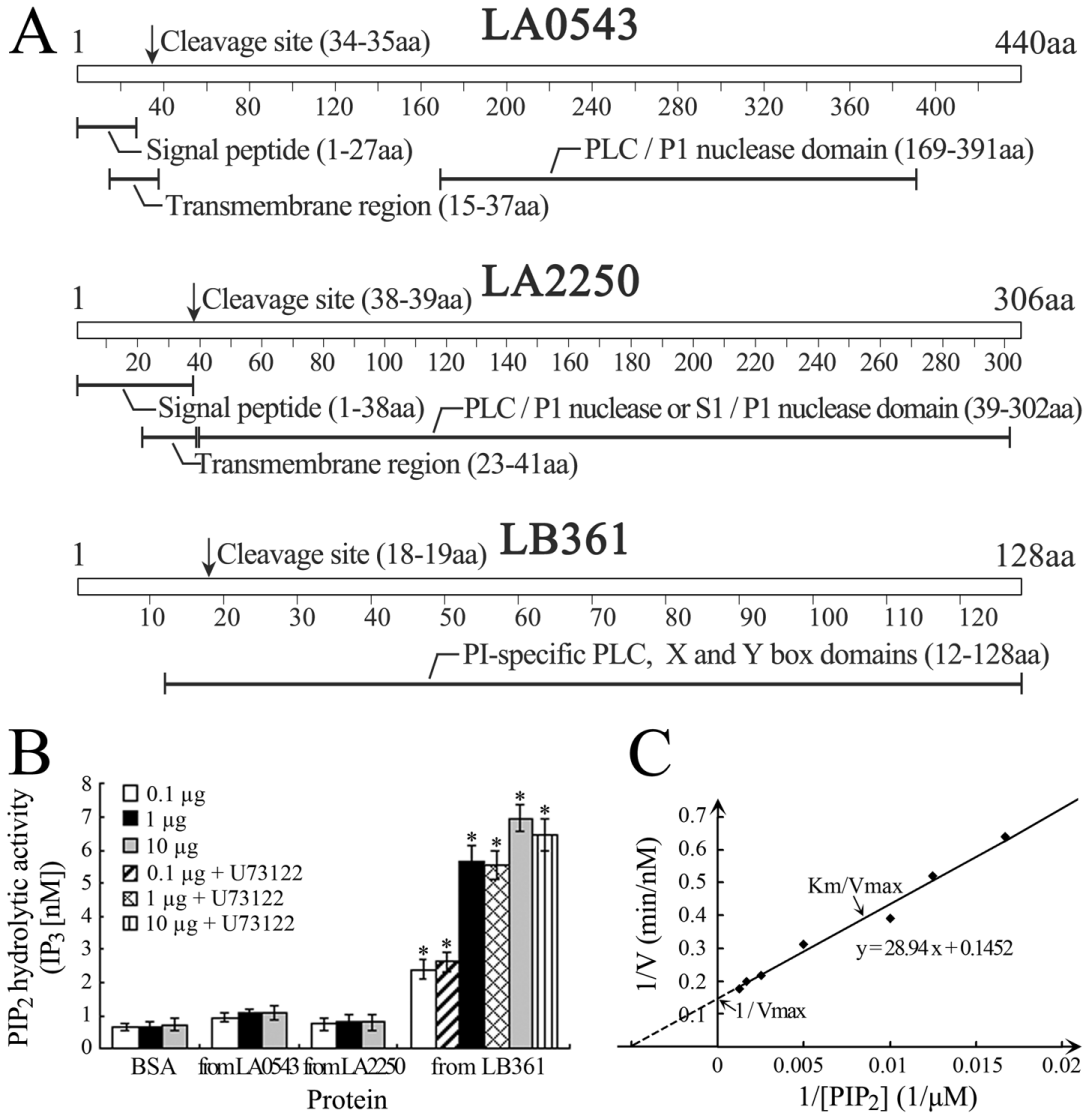
PLC domains in leptospiral genes and PI-PLC activity of leptospiral proteins. (A). PLC domains in the LA0543, LA2250 and LB361 genes of *L. interrogans* strain Lai. PLC domains were analyzed with SWISS-MODEL and InterProScan software. Signal peptides and transmembrane regions were predicted with SignalP 3.0 and TMHMM 2.0 software. (B). PI-PLC activity of the recombinant proteins expressed by the LA0543, LA2250 and LB361 genes of *L. interrogans* strain Lai. PI-PLC ability to hydrolyze PIP_2_ into IP_3_ was detected by IP_3_ fluorescence polarization assay. The data show the means ± SD of three independent experiments. BSA was a blank control to monitor fluorescence background. U73122 is a mammalian cell PI-PLC inhibitor. *: *p*<0.05 *vs* both the blank control and the recombinant proteins expressed by the LA0543 and LA2250 genes. The statistically significant differences were determined by ANOVA variance analysis plus Dunnett’s multiple comparison test. (C). Km and Kcat values of the recombinant protein of LB361 gene (rL-PI-PLE) determined by IP_3_ fluorescence polarization assay and double reciprocal Lineweaver-Burk plot. The concentration of rL-PI-PLC used was 1 µg, while the concentrations of PIP_2_ substrate used were 60, 80, 100, 200, 400, 600 or 800 µM.

The distribution of the LA0543, LA2250 and LB361 genes in different pathogenic or non-pathogenic *Leptospira* strains was examined as a way to predict the potential role of the genes in leptospiral pathogenicity. The PCR and sequencing data confirmed that all the seven tested pathogenic *L. interrogans* strains but not the two non-pathogenic *L. biflexa* strains belonging to different serogroups and serovars possessed the LA0543 and LB361 genes with high sequence identities, while the LA2250 gene was only detectable in genomic DNA from *L. interrogans* serogroup Icterohaemorrhagiae serovar Lai strain Lai (Figure S2A), suggesting a possible correlation of expression of the three genes with the pathogenicity of *L. interrogans*. On the other hand, our bioinformatic analysis revealed that there is a PLC domain in the amino acid sequences of the LA0543, LA2250 and LB361 genes of *L. interrogans* strain Lai (Figure 1A). However, only the LB361 gene was predicted as a PI-PLC due to its sequence containing X and Y box domains (Figure 1A). The formation of X and Y dimers has been shown to be necessary for enzymatic activity of PI-PLC in order to hydrolyze the PIP_2_ substrate [49].

### PI-PLC Activity of the Recombinant Protein Encoded by the LB361 Gene

The NPPC assay or IP_3_ fluorescence polarization assay was used to determine the PC-PLC or PI-PLC activity of recombinant proteins expressed by the LA0543, LA2250 and LB361 genes of *L. interrogans* strain Lai. The results of the NPPC assay demonstrated that none of the recombinant proteins expressed by the LA0543, LA2250 and LB361 genes of *L. interrogans* strain Lai expressed PC-PLC activity (data not shown). The IP_3_ fluorescence polarization assay confirmed that only the recombinant protein of the LB361 gene had PI-PLC activity to hydrolyze PIP_2_ into IP_3_ (Figure 1B), with the Km of 199 µM and Kcat of 8.566E-5 S^−1^ (Figure 1C). The product of the LB361 gene was designated as L-PI-PLC. However, U73122, a mammalian PI-PLC inhibitor [42], did not inhibit the PIP_2_ hydrolytic activity of rL-PI-PLC (Figure 1B).

### P_2_X_7_ Expression in J774A.1 and THP-1 Cells

**Figure 2:**
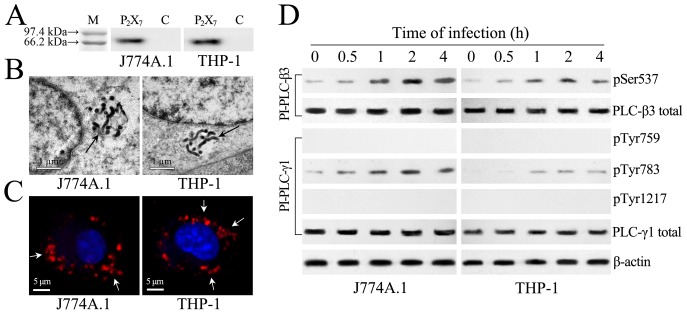
P_2_X_7_ expression, intracellular leptospire levels and infection-induced PI-PLC cellular phosphorylation. (A). P_2_X_7_ protein expression in J774A.1 and THP-1 cells. Lane M: protein marker. Lane P_2_X_7_: immunoblotting of P_2_X_7_ protein of J774A.1 or THP-1 cells with P_2_X_7_-IgG. Lane C: blank control. (B). Leptospires in J774A.1 or THP-1 cells viewed under the electron microscope after infection with *L. interrogans* strain Lai for 30 min. The arrows indicate the leptospires within phagocytotic vesicles in the cytosol of J774A.1 or THP-1 cells. (C). Leptospires in J774A.1 or THP-1 cells viewed under the laser confocal microscope after infection with *L. interrogans* strain Lai for 30 min. The small red spots correspond to the intracellular leptospires (pointed by the arrows), while the large blue plaques correspond to the cell nucleus. (D). Phosphorylation increase of PI-PLCs in J774A.1 and THP-1 cells during infection with *L. interrogans* strain Lai for the indicated times, determined by Western Blot assay. The images at “0 h” indicate the immunoblotting results of total PI-PLC-β3 or PI-PLC-γ1, pSer537 in PI-PLC-β3 and pTyr759, pTyr783 or pTyr1217 in PI-PLC-γ1 of the J774A.1 or THP-1 cells before infection.

Extracellular Ca2^+^ influx through membrane calcium channels can cause the increase of [Ca^2+^]i. Until now, at least fifteen calcium channels in mammalian cells have been identified [50]. However, among the four voltage-gated and seven receptor-gated calcium channels tested, only P_2_X_7_, a receptor-gated calcium channel, was detectable in both J774A.1 and THP-1 cells by Western Blot assay (Figure 2A), This result indicates that P_2_X_7_ is the unique calcium channel expressed by J774A.1 and THP-1 cells, and receptor-gated calcium channel blockers, such as SKF96365 [46], could be used to determine extracellular Ca^2+^ influx through the P_2_X_7_ calcium channel in J774A.1 or THP-1 cells during infection.

### Leptospire Levels in Macrophages and Activation of Cellular PI-PLC during Infection

Macrophages can phagocytose bacterial pathogens, and the phagocytosis results in activation of cellular PI-PLC [51]. In the present study, when J774A.1 and THP-1 cells were infected with *L. interrogans* strain Lai for 30 min, leptospires in the two macrophage types could be observed by electron microscopy (Figure 2B) and confocal microscopy (Figure 2C). Compared to the J774A.1 and THP-1 cells before infection, the phosphorylation levels at the serine 537 (pSer537) on PI-PLC-β3 and tyrosine 783 (pTyr783) on PI-PLC-γ1 of the two leptospire-infected macrophages were significantly increased (Figure 2D). However, phosphorylation at the Tyr759 and Tyr1217 of PI-PLC-γ1 was undetectable. The data suggest that PI-PLC-β3 and PI-PLC-γ1 of macrophages play a role in [Ca^2+^] elevation in macrophages during infection.

### Elevation of LB361-mRNA Levels during Infection

**Figure 3:**
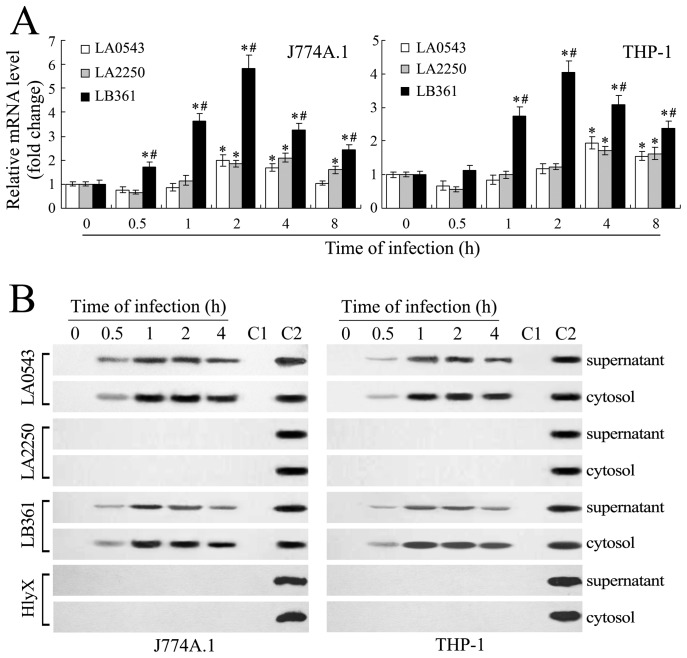
Increase in LB361 gene expression and L-PI-PLC secretion during infection. (A). Change of the LA0543-, LA2250- or LB361-mRNA level in *L. interrogans* strain Lai during infection of J774A.1 and THP-1 cells for the indicated times. Bars show the means ± SD of three independent experiments. The values at “0 h” indicate the relative LA0543-, LA2250- or LB361-mRNA level in the spirochete before infection, which was set as 1.0. *: *p*<0.05 *vs* the LA0543-, LA2250- or LB361-mRNA level in the spirochete before infection (0 h). ^#^: *p*<0.05 *vs* the LA0543- or LA2250-mRNA level in the spirochete during infection of J774A.1 and THP-1 cells. The statistically significant differences were determined by ANOVA variance analysis plus Dunnett’s multiple comparison test. (B). Secretion of the proteins encoded by the LA0543, LA2250 and LB361 genes of *L. interrogans* strain Lai during infection of J774A.1 and THP-1 cells for the indicated times. The supernatant samples are leptospire-free or cell-free liquids collected from the culture of the spirochete in EMJH medium (0 h) or from co-cultures of the spirochete with J774A.1 or THP-1 cells (0.5–4 h). The cytosol samples were collected from lysate of the J774A.1 or THP-1 cells before infection (0 h) or from leptospire-free lysates of the two macrophages after infection with the spirochete (0.5–4 h). Lane C1: blank control. Lane C2: immunoblotting result of the recombinant protein expressed by the LB361, LA0543 or LA2250 gene used as the positive control. HlyX is a cytoplasmic protein of the spirochete also used as a control.

The expression levels of a bacterial gene will increase if the gene is required by the pathogen during infection of hosts [19]. Our RT-qPCR analysis showed that the LB361-mRNA levels of *L. interrogans* strain Lai increased significantly during infection of J774A.1 or THP-1 cells with the maximal mRNA levels (4.04 or 5.83 fold increase) observed at the 2 h of post-infection (Figure 3A). Our previous microarray detection results showed the increase in LA0543-mRNA levels, the decrease in LA2250-mRNA levels, but no significant change in LB361-mRNA levels in the spirochetes during infection of macrophages [18]. However, RT-qPCR is more sensitive and accurate than microarray analysis. Therefore, the RT-qPCR data suggest that the LB361 gene could play a role in infection by the spirochete.

### External Secretion of L-PI-PLC in Macrophages during Infection

External secretion is necessary for L-PI-PLC to play a functional role in host cells. Our Western Blot assay demonstrated that none of the proteins expressed by the LA0543, LA2250 and LB361 genes were detectable in the supernatants from culture of *L. interrogans* strain Lai in EMJH medium. When the spirochete was incubated with J774A.1 or THP-1 cells, the product of the LB361 gene (L-PI-PLC) was secreted into the supernatants of co-cultures and cytosol of macrophages (Figure 3B). However, HlyX, a leptospiral nonsecreted cytoplasmic protein that was used as the control, was undetectable in all the supernatant and cytosol samples (Figure 3B). Although the protein expressed by the LA0543 gene was also detectable in the supernatants from co-cultures and cytosol of leptospire-infected J774A.1 or THP-1 cells (Figure 3B), this protein did not exhibit PI-PLC or PC-PLC activity (Figure 1B). However, the protein encoded by the LA2250 gene was undetectable in both the supernatant and cytosol samples. The data suggest that the L-PI-PLC of *L. interrogans* could play a direct role in macrophages during infection.

### [Ca^2+^]i Elevation Due to Extracellular Ca^2+^ Influx and Intracellular Ca^2+^ Release

**Figure 4:**
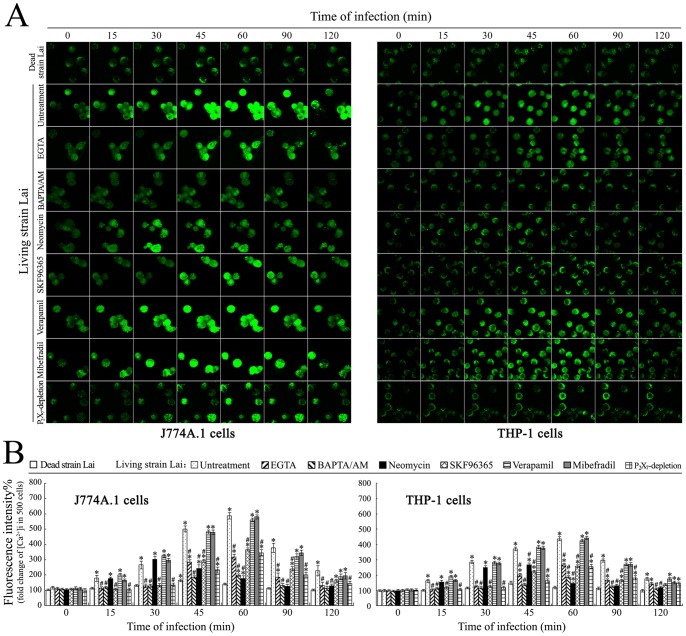
Elevation of [Ca^2+^]i in macrophages during infection with *L. interrogans*. (A). Elevation of [Ca^2+^]i in J774A.1 and THP-1 cells during infection with *L. interrogans* strain Lai for the indicated times, determined by laser confocal microscopy. The intensity of green fluorescence reflects the [Ca^2+^]i in macrophages. The dead strain Lai was obtained by heat killing the spirochete. EGTA is an extracellular Ca^2+^ chelator. BAPTA/AM is an intracellular free Ca^2+^ chelator. Neomycin is a blocker of IP_3_ production. SKF96365 is a receptor-gated calcium channel blocker. Verapamil or mibefradil is L-type or T-type voltage-gated calcium channel blocker. The images at “0 h” indicate the [Ca^2+^]i in J774A.1 and THP-1 cells before infection. (B). Statistical summary of the [Ca^2+^]i changes in the leptospire-infected macrophages. Data from experiments such as shown in A. Bars show the means ± SD of three independent experiments. The dead strain Lai corresponds to the heat-killed spirochete. The values at “0 h” indicate the [Ca^2+^]i in J774A.1 or THP-1 cells before infection. Five hundred cells were analyzed for each of the samples. *: *p*<0.05 *vs* the fluorescence intensity reflecting [Ca^2+^]i in the J774A.1 or THP-1 cells before infection (0 h). ^#^: *p*<0.05 *vs* the fluorescence intensity reflecting [Ca^2+^]i in the leptospire-infected J774A.1 or THP-1 cells untreated with the chelators or blockers. The statistically significant differences were determined by ANOVA variance analysis plus Dunnett’s multiple comparison test.

Extracellular Ca^2+^ influx and intracellular Ca^2+^ release are the most common ways to increase [Ca^2+^]i [20]. Compared to the [Ca^2+^]i in uninfectedJ774A.1 cells and THP-1 cells, the [Ca^2+^]i in the two macrophages increased rapidly after infection with *L. interrogans* strain Lai (Figure 4). The maximal [Ca^2+^]i in the leptospire-infected J774A.1 or THP-1 cells was observed at the 1 h of post-infection (Figure 4). Pretreatment with the extracellular Ca^2+^ chelator EGTA or IP_3_ production blocker neomycin caused a significant decrease of the [Ca^2+^]i elevation during infection (Figure 4). The receptor-gated calcium channel blocker SKF96365, but not the L-type or T-type voltage-gated calcium channel blockers (verapamil or mibefradil), could inhibit the increase in [Ca^2+^]i, and the P_2_X_7_-depleted J774A.1 or THP-1 cells also displayed a decrease in the [Ca^2+^]i elevation similar to the EGTA-pretreated macrophages (Figure 4). However, the heat-killed *L. interrogans* strain Lai did not induce an increase in [Ca^2+^]i in the macrophages. The data suggest that infection of *L. interrogans* induces an increase in [Ca^2+^]i in macrophages, and both extracellular Ca^2+^ influx through P_2_X_7_ receptor-gated calcium channel and intracellular Ca^2+^ release from endoplasmic reticulum contribute to the increase in [Ca^2+^]i.

### Reduced [Ca^2+^]i Elevation in Macrophages Infected with the ΔLB361 Mutant

**Figure 5:**
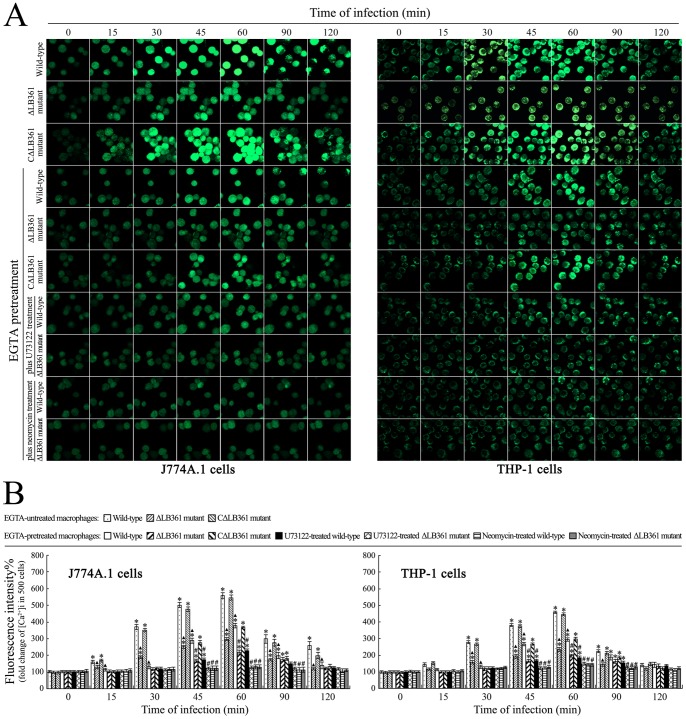
Reduced elevation of [Ca^2+^]i in ΔLB361 mutant-infected macrophages. (A). Change of [Ca^2+^]i in J774A.1 or THP-1 cells during infection with different leptospires for the indicated times determined by laser confocal microscopy.The intensity of green fluorescence reflects the [Ca^2+^]i in macrophages. The images at “0 h” indicate the [Ca^2+^]i in the J774A.1 and THP-1 cells before infection. EGTA is an extracellular Ca^2+^ chelator to block extracellular Ca^2+^ influx. U73122 is a mammalian cell PI-PLC inhibitor. Neomycin is a blocker of IP_3_ production. (B). Statistical summary of [Ca^2+^]i changes in the macrophages during infection with different leptospires. Data from experiments such as shown in A. Bars show the means ± SD of three independent experiments. The values at “0 h” indicate the [Ca^2+^]i in the J774A.1 and THP-1 cells before infection. Five hundred cells were analyzed for each of the samples. *: *p*<0.05 *vs* the fluorescence intensity reflecting [Ca^2+^]i in the J774A.1 or THP-1 cells before infection (0 h). ^▴^: *p*<0.05 *vs* the fluorescence intensity reflecting [Ca^2+^]i in the EGTA-untreated J774A.1 or THP-1 cells infected with wild-type *L. interrogans* strain Lai. ^#^: *p*<0.05 *vs* the fluorescence intensity reflecting [Ca^2+^]i in the EGTA-treated J774A.1 or THP-1 cells infected with wild-type *L. interrogans* strain Lai. The statistically significant differences were determined by ANOVA variance analysis plus Dunnett’s multiple comparison test.

Phospholipase C can regulate the [Ca^2+^]i by hydrolysis of PIP_2_ into IP_3_, an inducer of intracellular Ca^2+^ release from the endoplasmic reticulum [27]. Our fluorescent calcium indicator-based laser confocal microscopic examination revealed that the J774A.1 or THP-1 cells infected with the ΔLB361 mutant displayed a smaller [Ca^2+^]i elevation than the J774A.1 or THP-1 cells infected with wild-type *L. interrogans* strain Lai and the CΔLB361 mutant, but the [Ca^2+^]i in the two macrophages infected with the CΔLB361 mutant was similar to that in the two wild-type strain-infected macrophages (Figure 5). Pretreatment with the extracellular Ca^2+^ chelator EGTA plus the mammalian cell PI-PLC blocker U73122 caused a significant decrease of [Ca^2+^]i elevation in the ΔLB361 mutant-infected J774A.1 or THP-1 cells and a smaller decrease of [Ca^2+^]i elevation in the wild-type strain or CΔLB361 mutant-infected macrophages (Figure 5). The data suggest that both the product of LB361 gene and host cell PI-PLC contribute to the [Ca^2+^]i elevation in macrophages through intracellular Ca^2+^ release during infection with *L. interrogans*.

### Macrophage Death Due to [Ca^2+^]i Changes on Macrophage Death

**Figure 6:**
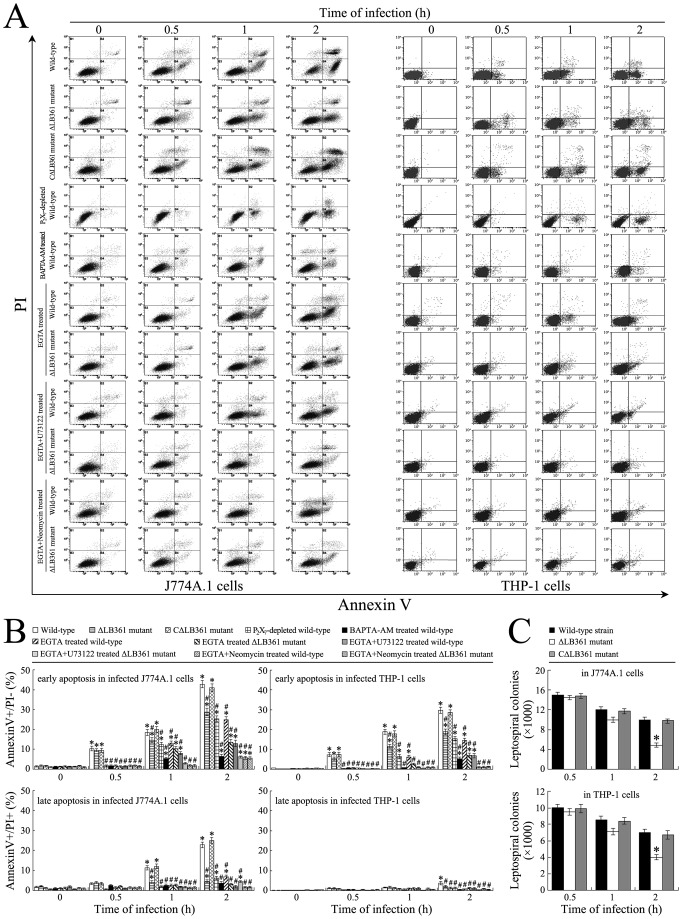
Macrophage death caused by *Leptospira*-induced [Ca^2+^]i elevation. (A). High [Ca^2+^]i-related apoptosis and necrosis in the J774A.1 or THP-1 cells during infection with different leptospires for the indicated times, determined by flow cytometry. The Annexin V^+^/PĪ cells represent early-apoptotic death while the Annexin V^+^/PI^+^ cells represent late-apoptotic or necrotic death. The images at “0 h” indicate the early or late apoptosis of the normal and P_2_X_7_-depleted J774A.1 or THP-1 cells before infection. EGTA is an extracellular Ca^2+^ chelator, BAPTA/AM is an intracellular free Ca^2+^ chelator. Neomycin is a blocker to inhibit IP_3_ production. U73122 is a mammalian cell PI-PLC inhibitor. (B). Statistical summary of early or late apoptotic/necrotic ratios in macrophages during infection with different leptospires. Data from experiments such as shown in A. Bars show the means ± SD of three independent experiments. The values at “0 h” indicate the early or late apoptosis of the normal or P_2_X_7_-depleted J774A.1 or THP-1 cells before infection. Five thousand cells were analyzed for each of the samples. *: *p*<0.05 *vs* the early apoptotic or late apoptotic/necrotic ratios in the J774A.1 or THP-1 cells before infection (0 h). ^#^: *p*<0.05 *vs* the early apoptotic or late apoptotic/necrotic ratios in the J774A.1 or THP-1 cells infected with wild-type *L. interrogans* strain Lai but untreated with the chelators, blockers or inhibitors. The statistically significant differences were determined by ANOVA variance analysis plus Dunnett’s multiple comparison test. (C). Diversity of viability of different leptospires in macrophages, determined by CFU enumeration. Bars show the means ± SD of three independent experiments. *: *p*<0.05 *vs* the CFUs of the CΔLB361 mutant and wild-type *L. interrogans* strain Lai from J774A.1 or THP-1 cells at the 2 h of post-infection. The statistically significant differences were determined by ANOVA variance analysis plus Dunnett’s multiple comparison test.

High [Ca^2+^]i had been shown to induce cell apoptosis or necrosis [52]. The flow cytometric analysis showed that the early-apoptotic and late-apoptotic/necrotic ratios of the ΔLB361 mutant-infected J774A.1 or THP-1 cells were significantly lower than that of the two macrophages infected with the CΔLB361 mutant or wild-type *L. interrogans* strain Lai (Figure 6A and B). When J774A.1 or THP-1 cells were pretreated with the chelator of intracellular Ca^2+^, BAPTA/AM, the extracellular Ca^2+^ chelator, EGTA, EGTA plus the IP_3_ production blocker neomycin, or the mammalian cell PLC inhibitor, U73122, there was a significance decrease of apoptosis or necrosis of the two macrophages infected with the different leptospires. The lowest apoptotic and necrotic ratios were obtained with BAPTA/AM-pretreated J774A.1 or THP-1 cells (Figure 6A and B). In particular, the apoptotic and necrotic ratios of P_2_X_7_-depleted J774A.1 or THP-1 cells were similar to those of the two EGTA-treated macrophages during infection. These results suggest that the [Ca^2+^]i elevation due to extracellular Ca^2+^ influx and intracellular Ca^2+^ release during infection with *L. interrogans* induces apoptosis or necrosis of macrophages.

### Attenuated Viability of ΔLB361 Mutant in Macrophages

Compared to the CΔLB361 mutant and wild-type *L. interrogans* strain Lai, the CFUs of the ΔLB361 mutant from the infected J774A.1 or THP-1 cells did not change significantly at the 0.5 or 1 h of post-infection, but displayed a significant decrease of CFU at the 2 h of post-infection (Figure 6C). The data suggest that the LB361 gene product contributes to survival of *L. inetrrogans* in macrophages.

### Elevation of IP_3_ Levels in LB361 Gene-transfected Macrophages

**Figure 7:**
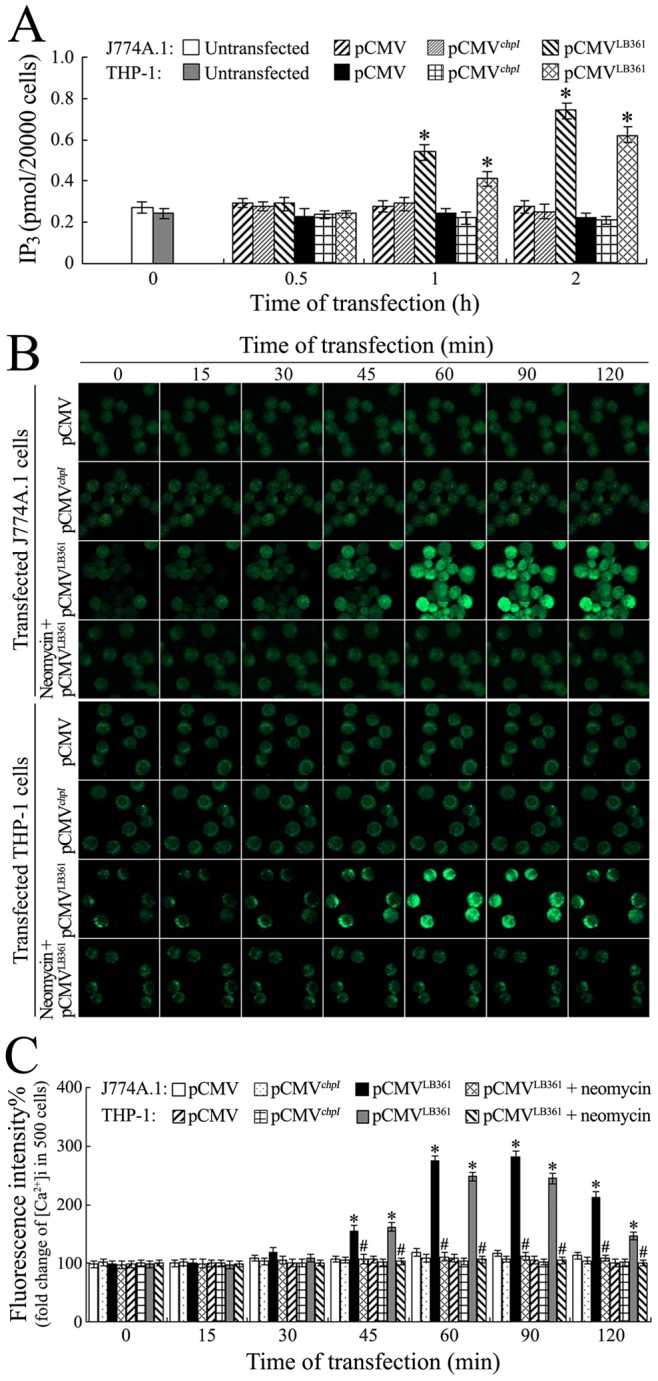
Elevation of IP_3_ levels and [Ca^2+^]i in LB361 gene-transfected macrophages. (A). Enhancement of IP_3_ levels in the LB361 gene-transfected macrophages, determined by IP_3_ fluorescence polarization assay. The values at “0 h” indicate the IP_3_ levels in the normal J774A.1 or THP-1 cells before transfection. *: *p*<0.05 *vs* the IP_3_ levels in the normal J774A.1 or THP-1 cells before transfection (0 h) and wild-type pCMV-Tag2C or *chpI* gene-transfected J774A.1 or THP-1 cells. The statistically significant differences were determined by ANOVA variance analysis plus Dunnett’s multiple comparison test. (B). Elevation of [Ca^2+^]i in the LB361 gene-transfected J774A.1 and THP-1 cells for the indicated times, determined by laser confocal microscopy. The intensity of green fluorescence reflects the [Ca^2+^]i in macrophages. The images at “0 min” indicate the [Ca^2+^]i in the normal J774A.1 and THP-1 cells before transfection. Neomycin is a blocker to inhibit IP_3_ production by. The wild-type pCMV-Tag2C or *chpI* gene-transfected J774A.1 or THP-1 cells were used to assess the influence of gene transfection or exogenous protein expression on the [Ca^2+^]i. (C). Statistical summary of the [Ca^2+^]i changes by laser confocal microscopy in the LB361 gene-transfected macrophages. Data from experiments such as shown in B. Bars show the means ± SD of three independent experiments. The values at “0 min” indicate the [Ca^2+^]i in the normal J774A.1 or THP-1 cells before transfection. Five hundred cells were analyzed for each of the samples. *: *p*<0.05 *vs* the fluorescence intensity reflecting [Ca^2+^]i in the normal J774A.1 or THP-1 cells before transfection (0 min) and wild-type pCMV-Tag2C or *chpI* gene-transfected J774A.1 or THP-1 cells. ^#^: *p*<0.05 *vs* the fluorescence intensity reflecting [Ca^2+^]i in the neomycin-untreated LB361 gene-transfected J774A.1 or THP-1 cells. The statistically significant differences were determined by ANOVA variance analysis plus Dunnett’s multiple comparison test.

PI-PLC hydrolyzes PIP_2_ to produce IP_3_ to cause the increase of intracellular IP_3_ [27]. The IP3 fluorescence polarization assay demonstrated that the IP_3_ levels in the LB361 gene-transfected J774A.1 or THP-1 cells were significantly higher than in the macrophages without transfection, or in the wild-type pCMV-Tag2C or *chpI* gene-transfected macrophages (Figure 7A). The data suggest that the product of the *L. interrogans* LB361 gene (L-PI-PLC) hydrolyzes PIP_2_ into IP_3_ in macrophages.

### L-PI-PLC-induced [Ca^2+^]i Elevation and Apoptosis of Macrophages

**Figure 8:**
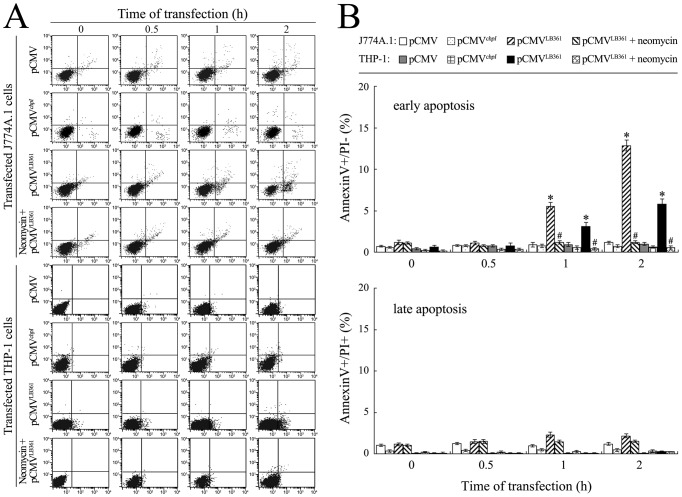
Macrophage apoptosis caused by LB361 gene transfection. (A). Apoptosis of the LB361 gene-transfected J774A.1 or THP-1 cells for the indicated times, determined by flow cytometry. The Annexin V^+^/PĪ cells represent early-apoptotic cells. The Annexin V^+^/PI^+^ cells represent late-apoptotic or necrotic cells. The images at “0 h” indicate the early or late apoptosis/necrosis of J774A.1 or THP-1 cells before transfection. The two macrophages were pretreated with EGTA to block extracellular Ca^2+^ influx. Neomycin is a blocker to inhibit IP_3_ production. (B). Statistical summary of the early-apoptotic ratios in the LB361 gene-transfected macrophages. Data from experiments such as shown in A. Bars show the means ± SD of three independent experiments. The values at “0 h” indicate the early-apoptosis or late-apoptosis/necrosis of J774A.1 or THP-1 cells before transfection. Five thousand cells were analyzed for each of the samples. *: *p*<0.05 *vs* the early-apoptotic ratios in the J774A.1 or THP-1 cells before transfection, and wild-type pCMV-Tag2C or *chpI* gene-transfected J774A.1 or THP-1 cells. ^#^: *p*<0.05 *vs* the early-apoptotic ratios in the LB361 gene-transfected but neomycin-untreated J774A.1 or THP-1 cells. The statistically significant differences were determined by ANOVA variance analysis plus Dunnett’s multiple comparison test.

To obtain direct evidence that the LB361 gene product of *L. interrogans* strain Lai can cause [Ca^2+^]i-dependent death of macrophages, we measured the [Ca^2+^]i and apoptosis/necrosis of the LB361 gene-transfected J774A.1 or THP-1 cells. The results showed that the LB361 gene-transfected J774A.1 or THP-1 cells displayed a detectable increase in [Ca^2+^]i compared to the two normal or wild-type pCMV-Tag2C-transfected macrophages, but the IP_3_ production blocker neomycin inhibited the [Ca^2+^]i elevation (Figure 7B and C). The LB361 gene-transfected J774A.1 or THP-1 cells displayed the maximal early-apoptotic ratio (12.84% or 5.82%) at the 2 h post-transfection, and the pretreatment with neomycin decreased the early-apoptotic ratios (Figure 8). However, the transfection with the leptospiral *chpI* gene in J774A.1 or THP-1 cells did not affect the [Ca^2+^]i and early-apoptotic ratios (Figure 7 and 8). The data suggest that the LB361 gene product (L-PI-PLC) causes a mild [Ca^2+^]i increase, which promotes macrophage apoptosis.

## Discussion

Intracellular free Ca^2+^ is an important mediator of cell signaling pathways that regulate many physiological functions such as metabolism, protein secretion, cell division, phagocytosis, cell death, and muscle contraction [53]. In particular, Ca^2+^ also modulates cellular stress responses to environmental stimuli [54]. Infection can also be viewed as a source of stress for both microbial pathogens and their infected hosts. Several pathogenic bacteria, such as *L. monocytogenes*, *H. pylori*, *C. jejuni* and *B. abortus*, can stimulate an increase in [Ca^2+^]i in different host cells including macrophages [21]–[24]. The results of this study also found that infection with *L. interrogans* strain Lai induces a significant increase in [Ca^2+^]i in macrophages from murine or human origin.

Intracellular free Ca^2+^ concentration ([Ca^2+^]i) can increase due to extracellular Ca^2+^ influx through membrane calcium channels or intracellular Ca^2+^ release from endoplasmic reticulum [53]–[55]. The intracellular Ca^2+^ release occurs when the IP_3_-receptor (IP_3_R) on endoplasmic reticulum is ligated with cytoplasmic IP_3_ [27], [56]. So far, many different calcium channels that belong to voltage-gated or receptor-gated family have been characterized [47], [48], [50]. A previous study reported that mouse J774 macrophages express both P_2_X_4_ and P_2_X_7_ receptor-gated calcium channels [57]. However, J774A.1 and THP-1 cells used in this study only express P_2_X_7_ protein. In the present study, we demonstrated that pretreatment with EGTA, an extracellular Ca^2+^ chelator, or neomycin, a blocker of IP_3_ production [45], repressed the [Ca^2+^]i elevation in the J774A.1 and THP-1 cells during infection with *L. interrogans* strain Lai. Moreover, the receptor-gated calcium channel blocker (SKF96365), but not the voltage-gated Ca^2+^ channel blockers, could inhibit the *Leptospira*-induced [Ca^2+^]i elevation in the two macrophages, while the depletion of P_2_X_7_ also prevented the [Ca^2+^]i elevation during infection. The data imply that *L. interrogans* causes the increase in [Ca^2+^]i in the infected macrophages due to extracellular Ca^2+^ influx through P_2_X_7_ calcium channel and intracellular Ca^2+^ release from endoplasmic reticulum.

PI-PLC can increase cellular [Ca^2+^]i after hydrolysis of PIP_2_ into IP_3_, which binds to IP_3_R to trigger Ca^2+^ release from endoplasmic reticulum into cytosol [27], [56], [58]. Although an earlier report revealed that some bacterial proteins exhibited PLC activity [59], so far only one PI-PLC from *L. monocytogenes* has been well characterized [28]. In the present study, among the three PLC domain-containing genes of *L. interrogans* strain Lai, only the product of the LB361 gene (L-PI-PLC) displayed PI-PLC activity. More importantly, the increased expression and external secretion of L-PI-PLC were confirmed during infection of J774A.1 and THP-1 cells with the spirochete. Moreoever, all the seven tested *L. interrogans* strains belonging to different serogroups and serovars, but not the non-pathogenic *L. interrogans* strains, possess the LB361 gene. These data suggest that the product of the LB361 gene is a PI-PLC that is required by *L. interrogans* during infection.

The host cell PI-PLC during infection with some bacterial pathogens, such as *Yersinia enterocolitica* and *Mycobacterium tuberculosis*, can be activated by phosphorylation [60], [61]. In the present study, the phosphorylation elevation of PI-PLC-β3 and PI-PLC-γ1 in the J774A.1 or THP-1 cells was also observed during infection with *L. interrogans* strain Lai, and pretreatment with a cellular PI-PLC blocker (U73122) also dampened the elevation of [Ca^2+^]i and IP_3_ levels in the two macrophages during infection with the spirochete. These data imply that the cellular PI-PLCs also play a role in [Ca^2+^]i elevation in macrophages during infection with *L. interrogans*.

Infection-induced macrophage death is viewed as a common strategy used by different pathogens to evade the host immune response [62]. Since macrophages are the only phagocytes that can kill phagocytosed intracellular leptospires in the absence of *Leptospira-*specific antibodies [8]–[12], *Leptospira*-induced macrophage death is important for the ability of the spirochetes to survive in infected hosts [15], [16]. Previous studies reported that a mild increase in [Ca^2+^]i induces cell apoptosis through a mitochondrion-dependent apoptotic pathway, while large [Ca^2+^]i increases cause cell necrosis through activation of Ca^2+^-dependent phospholipases and proteinases [25], [52], [63]. Our results showed that infection of murine and human macrophages with *L. interrogans* strain Lai caused both apoptosis and necrosis. However, blockage of extracellular Ca^2+^ influx and/or intracellular Ca^2+^ release favored apoptosis over necrosis. In addition, the LB361 gene-transfected macrophages only exhibited cell apoptosis, which may be due to L-PI-PLC causing a mild increase in [Ca^2+^]i. Taken together, our data show that infection with *L. interrogans* induced an increase in [Ca^2+^]i and [Ca^2+^]i-dependent apoptosis and necrosis of macrophages, and the LB361 gene product is a novel leptospiral PI-PLC that contributes to [Ca^2+^]i-dependent macrophage death.

## Supporting Information

Figure S1 **Strategy for generation of ΔLB361 and CΔLB361 mutants.** See Materials S1 for details.
(TIF)

Figure S2 **Amplification and expression of LA0543, LA2250 and LB361 genes.** (A). Amplification of LA0543, LA2250 and LB361 genes in different leptospiral strains. Lane M: DNA marker. Lane 1: blank controls. Lanes 2 to 8: amplicoms of the LA0543 gene (1320 bp) and LB361 gene (384 bp) from pathogenic *L. interrogans* serovar Lai strain Lai, serovar Grippotyphosa strain Lin-6, serovar Autumnalis strain Lin-4, serovar Pomona strain Luo, serovar Hebdomadis strain 56069, serovar Australis strain 65-9 and serovar Canicola strain Lin, respectively, but only *L. interrogans* strain Lai provided an amplicom (918 bp) of LA2250 gene, Lanes 9 and 10: no amplification products of the LA0543, LA2250 and LB361 genes from non-pathogenic *L. biflexa* serovar Patoc strain Patoc-1 and serovar Adamana strain CH-11. (B). Expression of LA0543, LA2250 and LB361 genes of *L. interrogans* strain Lai and purification of recombinant proteins. Lane M: protein marker. Lane 1: blank control of wild-type pET42a-transformed *E. coli* BL21DE3. Lanes 2 to 4: the recombinant proteins expressed by LA0543, LA2250 and LB361 genes, respectively. Lanes 5 to 7: the purified recombinant proteins of LA0543, LA2250 and LB361 genes by Ni-NTA affinity chromatography, respectively.
(TIF)

Figure S3 **Confirmation of ΔLB361 and CΔLB361 mutants by PCR and sequencing.** (A). PCR results for identification of the ΔLB361 mutant. Lane M: DNA marker. Lane 1: blank control. Lane 2: amplicon (2668 bp) of the 5′arm-kan-3′arm (2428 bp) plus two extending regions (120 bp each) from the ΔLB361 mutant. Lane 3: amplicon (1981 bp) of the 5′arm-LB361-3′arm (1741 bp) plus two extending regions (120 bp each) form wild-type *L. interrogans* strain Lai. (B). PCR results for identification of the CΔLB361 mutant. Lane M: DNA marker. Lane 1: blank control. Lane 2: amplicon (3228 bp) of the 5′arm-LB361-spc-3′arm segment (2988 bp) plus two extending regions (120 bp each) from the CΔLB361 mutant. Lane 3: amplicon (2668 bp) of the 5′arm-kan-3′arm (2428 bp) plus two extending regions (120 bp each) from the ΔLB361 mutant. Lane 4: amplicon (1981 bp) of the 5′arm-LB361-3′arm (1741 bp) plus two extending regions (120 bp each) form wild-type *L. interrogans* strain Lai. (C). Schematic diagram of sequencing result of the ΔLB361 mutant. The positions of PCR primers used are marked below. (D). Schematic diagram of sequencing result of the CΔLB361 mutant. The positions of PCR primers used are marked below.
(TIF)

Figure S4 **Confirmation of ΔLB361 and CΔLB361 leptospiral mutants and LB361 or** ***chpI*** **gene-transfected and P_2_X_7_-depleted macrophages.** (A). Expression of LB361 gene in the ΔLB361 and CΔLB361 mutants determined by Western Blot assay. Lane 1: the protein expressed by LB361 gene in wild-type *L. interrogans* strain Lai. Lane 2: no LB361 gene-encoding protein detectable in the ΔLB361 mutant. Lane 3: the protein expressed by LB361 gene in the CΔLB361 mutant. Lane 4: blank control. (B). Expression of the LB361 gene in the LB361 gene-transfected macrophages determined by Western Blot assay. Lane 1 or 3: the protein expressed by LB361 gene in the LB361 gene-transfected J774A.1 or THP-1 cells. Lane 2 or 4: no LB361 gene-encoding protein detectable in the normal J774A.1or THP-1 cells without transfection. Lane 5: blank control. (C). Expression of ChpI protein in the *chpI* gene-transfected macrophages determined by Western Blot assay. Lane 1 or 3: the expressed ChpI protein in the *chpI* gene-transfected J774A.1 or THP-1 cells. Lane 2 or 4: no ChpI protein detectable in the normal J774A.1or THP-1 cells without transfection. Lane 5: blank control. (D). Absence of P_2_X_7_ protein in the P_2_X_7_-depleted macrophages determined by Western Blot assay. Lane 1 or 3: no P_2_X_7_ protein detectable in the P_2_X_7_-depleted J774A.1 or THP-1 cells. Lane 2 or 4: the P_2_X_7_ protein expressed by the normal J774A.1 or THP-1 cells without transfection. Lane 5: blank control. (E). Expression of the LB361 gene product in the LB361 gene-transfected J774A.1 or THP-1 cells, determined by laser confocal microscopy. The small green spots correspond to the protein expreesed by the LB361 gene in the transfected J774A.1 or THP-1 cells. The large blue plaques correspond to the cell nucleus. The images at “0″ h indicate the results of laser confocal microscopic examination of normal J774A.1 or THP-1 cells before LB361 gene transfection.
(TIF)

Table S1 Sequences of the primers used in this study.
(DOC)

Materials S1 **Detection and expression of LA0543, LA2250 and LB361 genes, and generation and identification of LB361 gene deletion and transfection.**
(DOC)
