# Supplementary figures and images for: Identification of Leptospira interrogans Phospholipase C as a Novel Virulence Factor Responsible for Intracellular Free Calcium Ion Elevation during Macrophage Death
Source: PLoS One. 2013 Oct 4;8(10):e75652. doi: 10.1371/journal.pone.0075652 (PMC3790881; doi:10.1371/journal.pone.0075652)

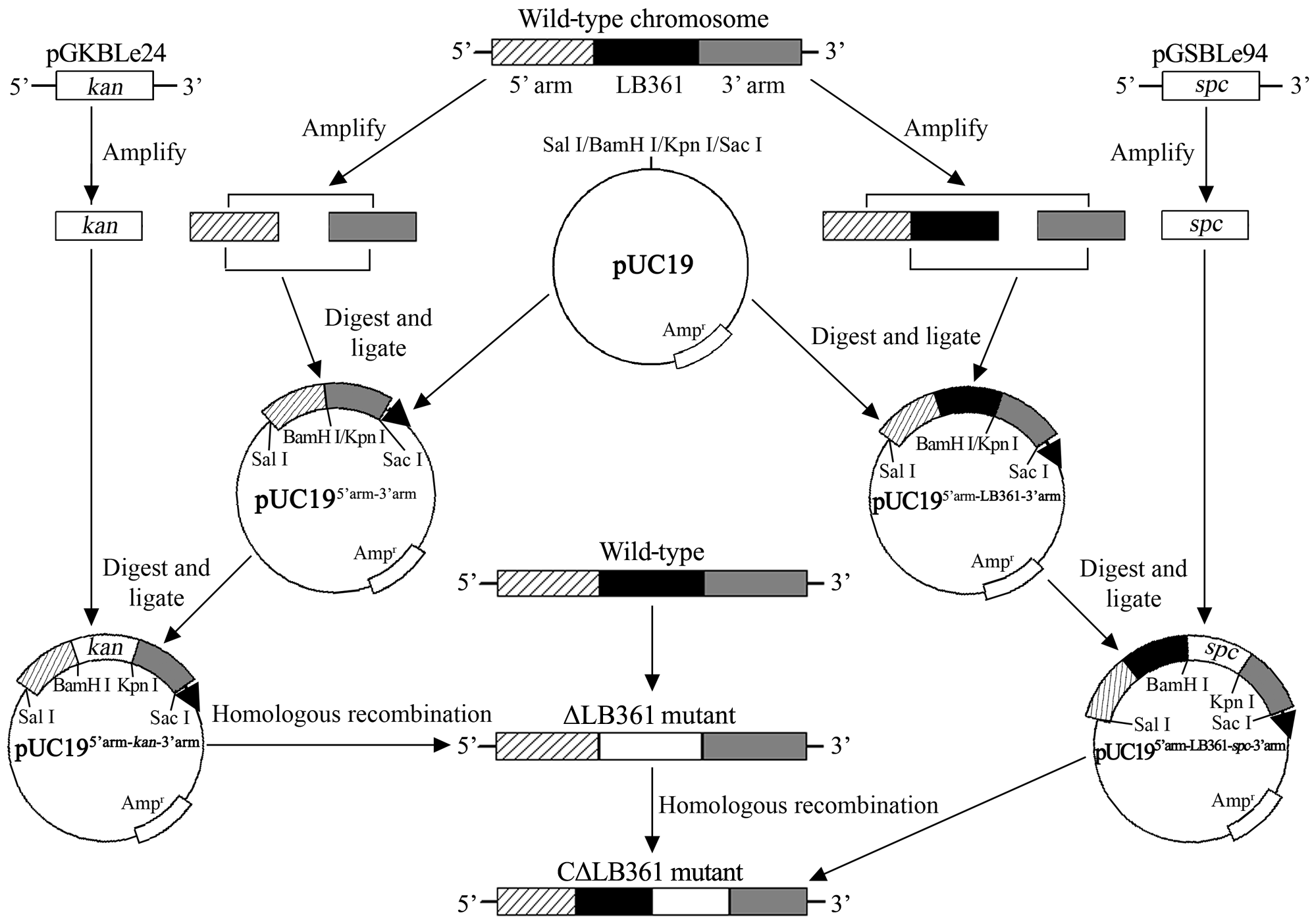

Supplement: Figure S1 — Strategy for generation of ΔLB361 and CΔLB361 mutants. See Materials S1 for details. (TIF) [file pone.0075652.s001.tif]

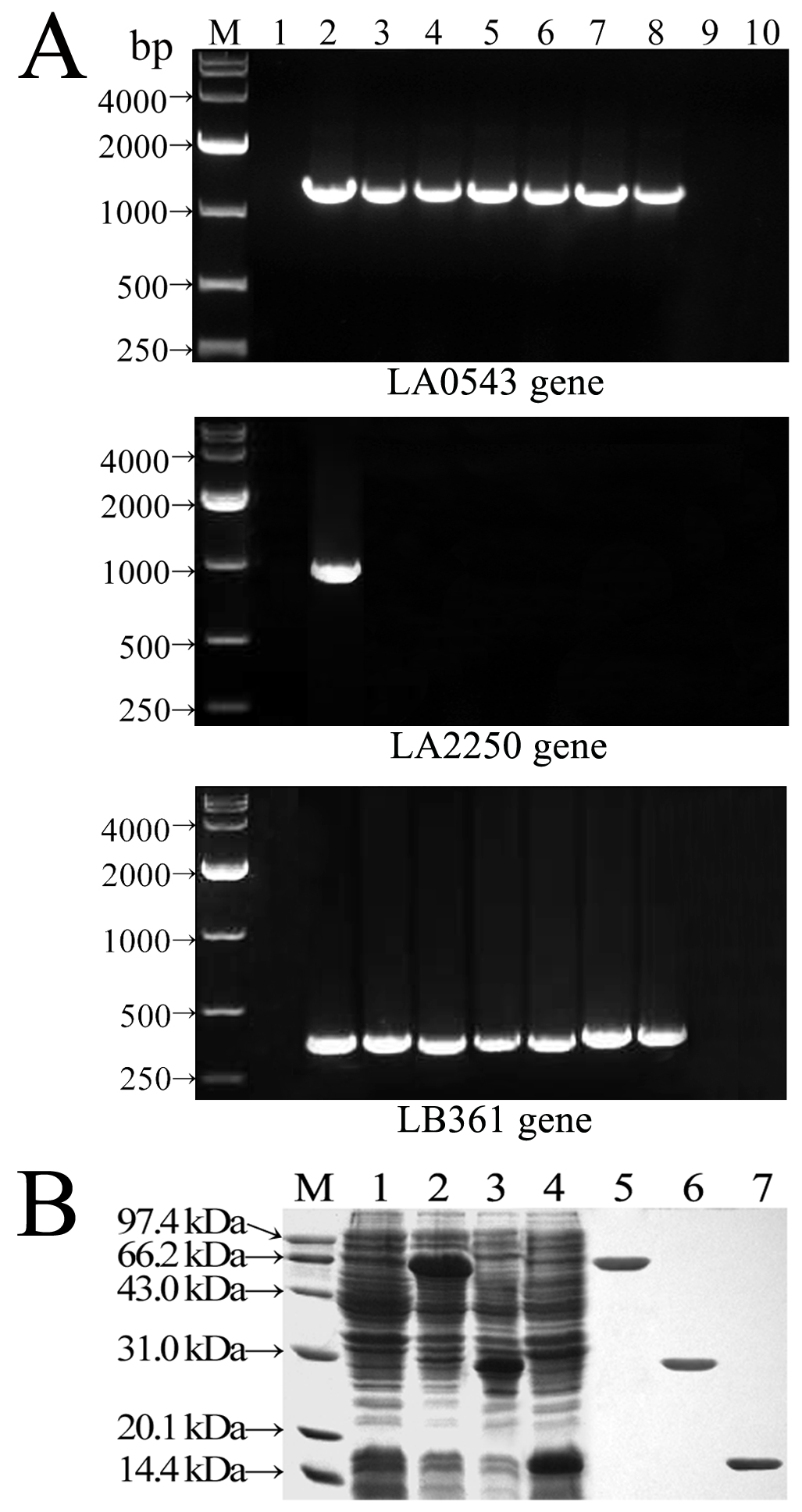

Supplement: Figure S2 — Amplification and expression of LA0543, LA2250 and LB361 genes. (A). Amplification of LA0543, LA2250 and LB361 genes in different leptospiral strains. Lane M: DNA marker. Lane 1: blank controls. Lanes 2 to 8: amplicoms of the LA0543 gene (1320 bp) and LB361 gene (384 bp) from pathogenic L. interrogans serovar Lai strain Lai, serovar Grippotyphosa strain Lin-6, serovar Autumnalis strain Lin-4, serovar Pomona strain Luo, serovar Hebdomadis strain 56069, serovar Australis strain 65-9 and serovar Canicola strain Lin, respectively, but only L. interrogans strain Lai provided an amplicom (918 bp) of LA2250 gene, Lanes 9 and 10: no amplification products of the LA0543, LA2250 and LB361 genes from non-pathogenic L. biflexa serovar Patoc strain Patoc-1 and serovar Adamana strain CH-11. (B). Expression of LA0543, LA2250 and LB361 genes of L. interrogans strain Lai and purification of recombinant proteins. Lane M: protein marker. Lane 1: blank control of wild-type pET42a-transformed E. coli BL21DE3. Lanes 2 to 4: the recombinant proteins expressed by LA0543, LA2250 and LB361 genes, respectively. Lanes 5 to 7: the purified recombinant proteins of LA0543, LA2250 and LB361 genes by Ni-NTA affinity chromatography, respectively. (TIF) [file pone.0075652.s002.tif]

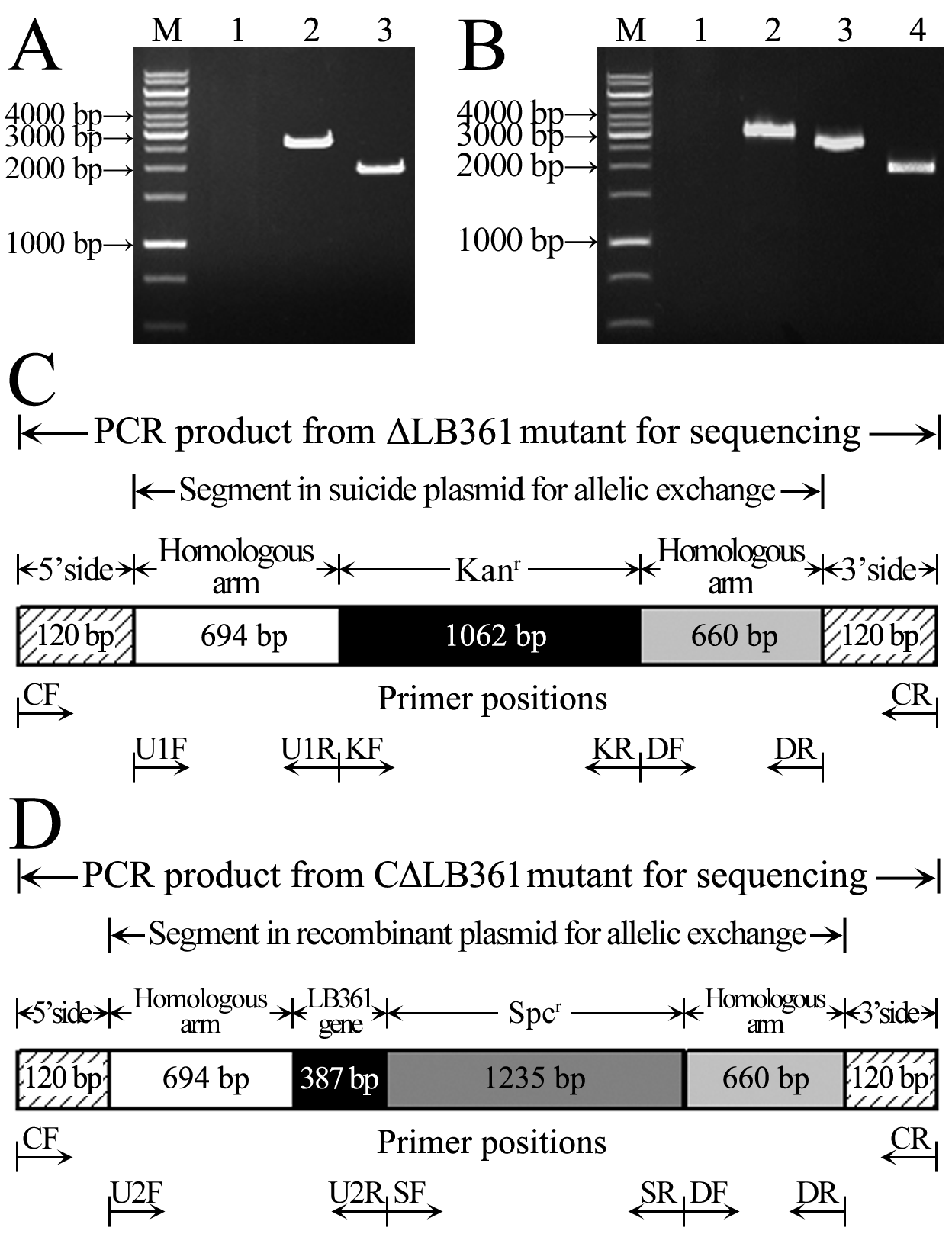

Supplement: Figure S3 — Confirmation of ΔLB361 and CΔLB361 mutants by PCR and sequencing. (A). PCR results for identification of the ΔLB361 mutant. Lane M: DNA marker. Lane 1: blank control. Lane 2: amplicon (2668 bp) of the 5′arm-kan-3′arm (2428 bp) plus two extending regions (120 bp each) from the ΔLB361 mutant. Lane 3: amplicon (1981 bp) of the 5′arm-LB361-3′arm (1741 bp) plus two extending regions (120 bp each) form wild-type L. interrogans strain Lai. (B). PCR results for identification of the CΔLB361 mutant. Lane M: DNA marker. Lane 1: blank control. Lane 2: amplicon (3228 bp) of the 5′arm-LB361-spc-3′arm segment (2988 bp) plus two extending regions (120 bp each) from the CΔLB361 mutant. Lane 3: amplicon (2668 bp) of the 5′arm-kan-3′arm (2428 bp) plus two extending regions (120 bp each) from the ΔLB361 mutant. Lane 4: amplicon (1981 bp) of the 5′arm-LB361-3′arm (1741 bp) plus two extending regions (120 bp each) form wild-type L. interrogans strain Lai. (C). Schematic diagram of sequencing result of the ΔLB361 mutant. The positions of PCR primers used are marked below. (D). Schematic diagram of sequencing result of the CΔLB361 mutant. The positions of PCR primers used are marked below. (TIF) [file pone.0075652.s003.tif]

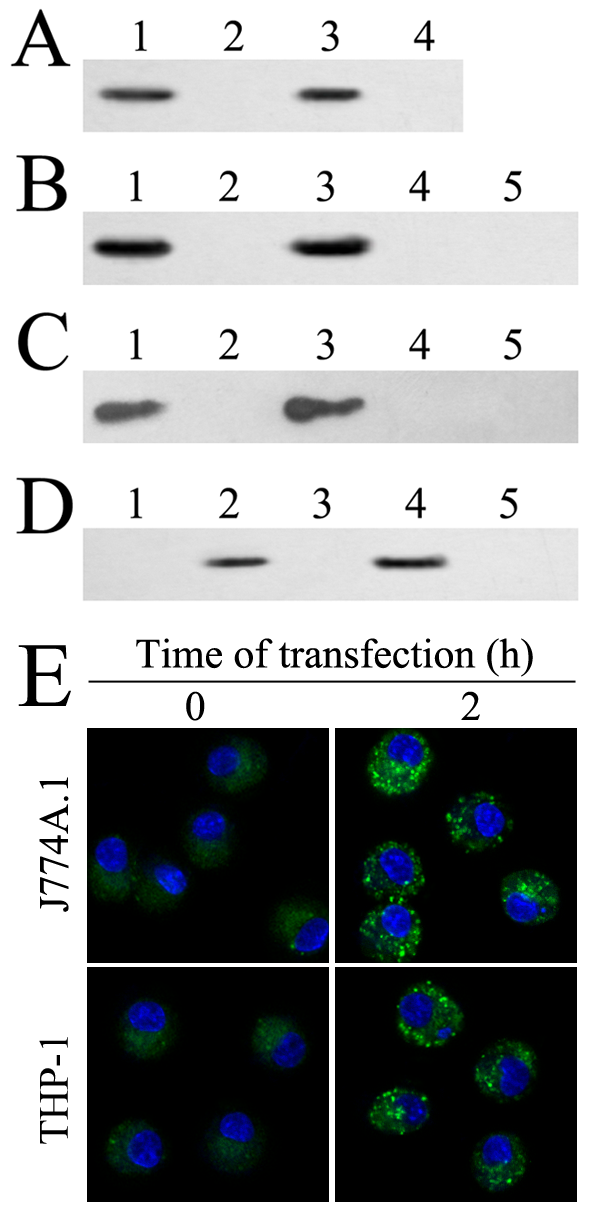

Supplement: Figure S4 — Confirmation of ΔLB361 and CΔLB361 leptospiral mutants and LB361 or chpI gene-transfected and P2X7-depleted macrophages. (A). Expression of LB361 gene in the ΔLB361 and CΔLB361 mutants determined by Western Blot assay. Lane 1: the protein expressed by LB361 gene in wild-type L. interrogans strain Lai. Lane 2: no LB361 gene-encoding protein detectable in the ΔLB361 mutant. Lane 3: the protein expressed by LB361 gene in the CΔLB361 mutant. Lane 4: blank control. (B). Expression of the LB361 gene in the LB361 gene-transfected macrophages determined by Western Blot assay. Lane 1 or 3: the protein expressed by LB361 gene in the LB361 gene-transfected J774A.1 or THP-1 cells. Lane 2 or 4: no LB361 gene-encoding protein detectable in the normal J774A.1or THP-1 cells without transfection. Lane 5: blank control. (C). Expression of ChpI protein in the chpI gene-transfected macrophages determined by Western Blot assay. Lane 1 or 3: the expressed ChpI protein in the chpI gene-transfected J774A.1 or THP-1 cells. Lane 2 or 4: no ChpI protein detectable in the normal J774A.1or THP-1 cells without transfection. Lane 5: blank control. (D). Absence of P2X7 protein in the P2X7-depleted macrophages determined by Western Blot assay. Lane 1 or 3: no P2X7 protein detectable in the P2X7-depleted J774A.1 or THP-1 cells. Lane 2 or 4: the P2X7 protein expressed by the normal J774A.1 or THP-1 cells without transfection. Lane 5: blank control. (E). Expression of the LB361 gene product in the LB361 gene-transfected J774A.1 or THP-1 cells, determined by laser confocal microscopy. The small green spots correspond to the protein expreesed by the LB361 gene in the transfected J774A.1 or THP-1 cells. The large blue plaques correspond to the cell nucleus. The images at “0″ h indicate the results of laser confocal microscopic examination of normal J774A.1 or THP-1 cells before LB361 gene transfection. (TIF) [file pone.0075652.s004.tif]
