# Supplementary material for: Identification of Leptospira interrogans Phospholipase C as a Novel Virulence Factor Responsible for Intracellular Free Calcium Ion Elevation during Macrophage Death
Source: PLoS One. 2013 Oct 4;8(10):e75652. doi: 10.1371/journal.pone.0075652 (PMC3790881; doi:10.1371/journal.pone.0075652)
Supplement: Table S1 — Sequences of the primers used in this study. (DOC) [file pone.0075652.s005.doc]

Table S1. Sequences of the primers used in this study

| Primer | Sequence (5’ to 3’) | Target | Size (bp) |
| --- | --- | --- | --- |
| LA0543 | F: atgatcaaaaatcaaaaaattg | LA0543 gene detection | 1320 |
|  | R: ATTTTTATGATTTGAATCTTG |  |  |
| LA2250 | F: Atgcttgtatttcaagttgta | LA2250 gene detection | 918 |
|  | R: TTCAGAACCAGGCTTAGAA |  |  |
| LB361 | F: ATGTTTATGGCTTCTAAAGTGA | LB361 gene detection | 384 |
|  | R: ACGTTTTGGGGCTTTTTTAG |  |  |
| LA0543-1 | F: GCCGGATCC(BamH I)CTCATTATGGATCAAATATTA | LA0543 gene expression | 1218 |
|  | R: GCCCTCGAG(Xho I)ATTTTTATGATTTGAATCTTG |  |  |
| LA2250-1 | F: GCCCATATG(Nde I)TGGGAAGGGCATCGTACA | LA2250 gene expression | 798 |
|  | R: GCCCTCGAG(Xho I)TTCAGAACCAGGCTTAGAA |  |  |
| LB361-1 | F: GCCCATATG(Nde I)TTTATGGCTTCTAAAGTGA | LB361 gene expression | 384 |
|  | R: GCCCTCGAG(Xho I)ACGTTTTGGGGCTTTTTTAG |  |  |
| LA0543-2 | F: TCCAACAGCCTTATTTCCCTG | LA0543-mRNA detection | 172 |
|  | R: TTCCCAAACTGCAAACTCGTC |  |  |
| LA2250-2 | F: TGACGAATCCTACCCATGACG | LA2250-mRNA detection | 181 |
|  | R: TCCGCAGTATGTAAAGGTTGATGTA |  |  |
| LB361-2 | F: GGCTTCTAAAGTGAAACGGTCA | LB361-mRNA detection | 179 |
|  | R: GATCAAAGTCCAACGGATTGTC |  |  |
| 16S | F: CTTTCGTGCCTCAGCGTCAGT | I6S rRNA as the internal | 145 |
|  | R: CGCAGCCTGCACTTGAAACTA | reference in RT-qPCR |  |
| U1 | F: CGCGTCGAC(Sal I)tttacatttatacggttttaa | 5’arm for LB361 gene | 694 |
|  | R: CGCGGATCC(BamH I)AGATTTTCAACTGCGGCTTGA | deletion |  |
| K | F: CGCGGATCC(BamH I)ATCGGCTCCGTCGATACTATG | Kan^r^ segment for LB361 | 1062 |
|  | R: CGCGGTACC(Kpn I)CATCAGAGTATGGACAGTTGC | gene deletion |  |
| D | F: CGCGGTACC(Kpn I)aaaagccctgtgctgactaca | 3’arm for LB361 gene | 660 |
|  | R: CGCGAGCTC(Sac I) AACTTTTTAGCTTCTATTTTC | deletion or complementation |  |
| U2 | F: CGCGTCGAC(Sal I)tttacatttatacggttttaa | 5’arm-LB361 segment for | 1081 |
|  | R: CGCGGATCC(BamH I)TTAACGTTTTGGGGCTTTTTTAGA | LB361 gene complementation |  |
| S | F: CGCGGATCC(BamH I)aacgcgtcccgagcttcaagg | Spc^r^ segment for LB361 | 1235 |
|  | R: CGCGGTACC(Kpn I)AACGCGTAAAGTAAGCACCTG | gene complementation |  |
| C | F: tttgaattcagatactgttgt | Confirmation of ΔLB361 | 2668 |
|  | R: TGCTACAAATTCAAAATTTAC | and CΔLB361 mutants | 3228 |
| T | F: GCCGGATCC(BamH I)ATGTTTATGGCTTCTAAAGTG | LB361 gene transfection | 390 |
|  | R: GCCCTCGAG(Xho I)TTAACGTTTTGGGGCTTTTTTAG |  |  |

F: forward primer. R: reverse primer. The underlined areas indicate the sites of endonucleases.
