# Supplementary material for: Identification of Leptospira interrogans Phospholipase C as a Novel Virulence Factor Responsible for Intracellular Free Calcium Ion Elevation during Macrophage Death
Source: PLoS One. 2013 Oct 4;8(10):e75652. doi: 10.1371/journal.pone.0075652 (PMC3790881; doi:10.1371/journal.pone.0075652)
Supplement: Materials S1 — Detection and expression of LA0543, LA2250 and LB361 genes, and generation and identification of LB361 gene deletion and transfection. (DOC) [file pone.0075652.s006.doc]

# Identification of *Leptospira interrogans* phospholipase C as a novel virulence factor responsible for intracellular free calcium ion elevation during macrophage death

Jing-Fang Zhao^1,2,3∆^, Hong-Hu Chen^2∆^, David M. Ojcius^4^, Xin Zhao^2^, Dexter Sun^5^, Yu-Mei Ge^1,2^, Lin-Li Zheng^2^, Xu’ai Lin^1,2^, Lan-Juan Li^1▲^, Jie Yan^1,2▲^

**Supplementary Materials**

**Materials and Methods**

**Leptospiral strains used in this study**

Seven pathogenic *L. interrogans* strains, serogroup Icterohaemorrhagiae serovar Lai strain Lai, serogroup Grippotyphosa serovar Grippotyphosa strain Lin-6, serogrouop Autumnalis serovar Autumnalis strain Lin-4, serogroup Pomona serovar Pomona strain Luo, serogroup Hebdomadis serovar Hebdomadis strain 56069, serogroup Australis serovar Australis strain 65-9, and serogroup Canicola serovar Canicola strain Lin (the prevalent *Leptospira* strain in China) officially served as the standard strains in serological examination for leptospirosis diagnosis in Chinese leptospirosis patients [1], and two non-pathogenic *L. biflexa* strains, serogroup Semmaranga serovar Patoc strain Patoc-1 and serogroup Andamana serovar Adamana strain CH-11, were used in this study. All the leptospiral strains were provided by the National Institute for Control of Pharmaceutical and Biological Products of China.

**Primers**

The primers used in this study were synthesized by Invitrogen Co. in Shanghai, China. The sequences of the primers are shown in Table S1.

**Detection of LA0543, LA2250 and LB361 genes in different leptospiral strains**

Genomic DNAs of the seven *L. interrogans* strains and two *L. biflexa* strains were extracted using a Bacterial Genomic DNA MiniPrep Kit (Axygen, USA). Three separate PCRs were performed to amplify the entire LA0543, LA2250 and LB361 genes from the DNAs using the primers LA0543F/LA0543R, LA2250F/LA2250R and LB361F/LB361R (Table S1), respectively. The products were examined on 1.5% ethidium bromide pre-stained agarose gels after electrophoresis, and then cloned into pMD18-T using a T-A Cloning Kit (TaKaRa, China) for sequencing by Invitrogen Co.

**Recombinant expression of LA0543, LA2250 and LB361 genes**

The signal peptide sequence-absent LA0543 and LA2250 genes and entire LB361 gene of *L. interrogans* strain Lai were amplified by PCR using the primers LA0543-1F/LA0543-1R, LA2250-1F/LA2250-1R and LB361-1F/LB361-1R (Table S1), respectively. The products were cloned in pMD18-T to form pMD18-T^LA0543^, pMD18-T^LA2250^ or pMD18-T^LB361^ for sequencing. The recombinant pMD18-T plasmids and pET42a vector (Novagen, USA) were digested with Nde I or BamH I and Xho I endonucleases (TaKaRa). Each of the three gene segments was linked with the linearized pET42a using T4 DNA ligase (TaKaRa) to form recombinant expression vectors which transformed into *E. coli* BL21DE3 (Novagen) to generate *E. coli* BL21DE3^pET42a-LA0543^, *E. coli* BL21DE3^pET42a-LA2250^ and *E. coli* BL21DE3^pET42a-LB361^ [2]. The engineered bacteria were cultured in kanamycin-containing LB medium (Oxoid, UK) to express the recombinant proteins of LA0543, LA2250, and LB361 genes under induction of 0.5 mM isopropy-β-D-thiogalactoside (IPTG, Sigma, USA). The expression of recombinant proteins was examined by SDS-PAGE plus an Agarose Image Analyzer (Bio-Rad, USA).

**Extraction of recombinant proteins expressed by LA0543, LA2250 and LB361 genes**

Each of the expressed recombinant proteins of LA0543, LA2250 and LB361 genes of *L. interrogans* strain Lai was extracted using a Ni-NTA affinity chromatographic column (BioColor, China) according to the manufacturer’s protocol, and the purity of extracted recombinant proteins was examined by SDS-PAGE plus an Agarose Image Analyzer (Bio-Rad).

**Preparation of antisera and IgGs**

Rabbits were immunized intradermally on days 1, 7, 14 and 21 with each of the purified leptospiral recombinant proteins that were pre-mixed with Freund’s adjuvant. Fifteen days after the last immunization, the rabbit sera were collected to separate IgGs by ammonium sulfate precipitation plus a DEAE-52 column (Sigma) using 10 mM phosphate buffer (pH 7.4) for elution. The titers of antisera or IgGs with the corresponding recombinant proteins were determined by immunodiffusion test.

**Generation of LB361 gene-deleted or complemented mutants**

Plasmid pGKBLe24 containing a kanamycin-resistant cassette (Kan^r^) and plasmid pGSBLe94 containing a spectinomycin-resistant cassette (Spc^r^) were graciously provided by Dr. Mathieu Picardeau (Pasteur Institute, France). Plasmid pUC19, which had been used to delete the *mce* gene of *L. interrogans* and the *trpE* gene of *L. meyeri* [2,3], was used for LB361 gene deletion (ΔLB361) and complementation (CΔLB361) because only the recombinant protein of LB361 gene was confirmed to have PI-PLC activity. In the chromosomal DNA of *L. interrogans* strain Lai (GenBank accession No.: NC_004342), the LB361 gene is located at the 3’ end in an operon (5’-LB362-LB361-3’). For allelic exchange to generate a ΔLB361 mutant, three separate PCRs were performed to amplify a 694-bp 5’arm DNA segment that located upstream of LB361 gene and a 660-bp 3’arm DNA segment that located downstream of the gene from genomic DNA of the spirochete, and the Kan^r^ sequence from pGKBLe24 plasmid using the primers U1F/U1R, DF/DR and KF/KR (Table S1), respectively. The 5’arm segment was digested with Sal I and BamH I endonucleases (TaKaRa), and then cloned into the Sal I and BamH I sites in pUC19 to form a recombinant plasmid pUC19^5’arm^. Subsequently, the 3’ arm segment was digested with Kpn I and Sac I endonucleases (TaKaRa), and then inserted into the Kpn I and Sac I sites in pUC19^5’arm^ to form another recombinant plasmid pUC19^5’arm-3’arm^. The *kan* segment was digested with BamH I and Kpn I, and then inserted into the BamH I and Kpn I sites in pUC19^5’arm-3’arm^ to form a suicide plasmid pUC19^5’arm-kan-3’arm^ for sequencing. The alkali-denature of suicide plasmid and electrocompetence of *L. interrogans* strain Lai were performed as previously described [2]. The competent leptospires were mixed with 2 µg of the suicide plasmid DNA for electrotransformation (1.8 kV, 200 Ω, 25 μF pulse) on ice for 10 min. The mixture was added with 1 mL Korthof liquid medium for a 48-h incubation at 28°C and then transferred onto Korthof agar plates containing 50 µg ml^-1^ kanamycin (Sigma) for a 15-d incubation to screen out colonies of ΔLB361 mutant. To generate a CΔLB361 mutant, three separate PCRs were performed to amplify a 1081-bp 5’arm-LB361 segment and a 660-bp 3’arm segment from genomic DNA of the spirochete, and the Spc^r^ sequence from plasmid pGSBLe94 using the primers U2F/U2R, DF/DR and SF/SR (Table S1), respectively. The subsequent endonuclease digestion and ligation of the segments (5’arm-LB361-spc-3’arm), formation of a recombinant plasmid pUC19^5’arm-LB361-spc-3’arm^, transformation of the plasmid into the ΔLB361 mutant and selection of CΔLB361 mutant colonies with spectinomycin (Sigma) were similar to those in generation of the ΔLB361 mutant. Finally, individual antibiotic-resistant colonies of the ΔLB361 or CΔLB361 mutant were cultured in antibiotic-containing EMJH liquid medium before use. The steps to generate the ΔLB361 and CΔLB361 mutants are summarized in Figure S1.

**Identification of ΔLB361 and CΔLB361 mutants**

Morphology, motility and growth kinetics of the ΔLB361 and CΔLB361 mutants were examined under a dark-field microscope [4]. The deletion mutation in the ΔLB361 mutant and the complemented mutation in the CΔLB361 mutant were determined by PCR with the primers CF/CR (Table S1) and sequencing as described above. The ΔLB361 and CΔLB361 mutants were ultrasonically broken on ice, followed by a 500×g centrifugation at 4°C for 15 min to harvest supernatants. Using 1:200 diluted rabbit anti-LB361 gene product-IgG as the primary antibody and 1:3000 diluted HRP-conjugated goat anti-rabbit-IgG (Jackson ImmunoResearch, USA) as the secondary antibody, Western Blot assay was performed to detect the protein expressed by LB361 gene in the ΔLB361 or CΔLB361 mutant. In the assay, wild-type *L. interrogans* strain Lai was used as the control.

**Generation and identification of LB361 or *chpI* gene-transfected macrophages**

A prokaryote-eukaryote shuttle plasmid, pCMV-tag2C, was kindly provided by Dr. Tian-Hua Zhou in Department of Cytobiology, Zhejiang University School of Medicine, China. Initially, a PCR was performed to amplify the entire LB361 gene of *L. interrogans* strain Lai using the primers TF/TR (Table S1). The PCR product was digested with BamH I and Xho I endonucleases (TaKaRa), and then inserted into the BamH I and Xho I sites of pCMV-tag2C using T4 DNA ligase (TaKaRa) to form pCMV-tag2c^LB361^ for sequencing. J774A.1 or THP-1 cells were transfected with pCMV-Tag2C^LB361^ using a Lipofectamine 2000 Transfection Kit (invitrogen) or a Human Monocyte Nucleofector Kit (Lonza, Germany) in a Nucleofector-II (type AAD-1001, Lonza) and then maintained in 10%FCS RPMI-1640 at 37°C for 2 h for transfection stability according to the manufacturers’ protocols [5]. The two LB361 gene-transfected macrophages were lysed with 0.05% NaTDC-PBS [2], followed by a 500×g centrifugation at 4°C for 15 min to harvest supernatants. The protein expressed by LB361 gene in the LB361 gene-transfected macrophages were detected by Western Blot assay as above. Using 1:200 diluted rabbit-IgG against the recombinant protein of the LB361 gene as the primary antibody, 1:500 diluted FITC488-conjugated sheep anti-rabbit-IgG (Jackson ImmunoResearch) as the secondary antibody, and DAPI (Invitrogen) as the dye of cell nuclei, the expression efficiency of the LB361-gene product in the two transfected macrophages was further determined on a laser confocal microscope (type LSM510, Zeiss, Germany) as previously described [2]. To estimate the influence of exogenous protein expression on [Ca^2+^]i and viability of transfected macrophages, the *chpI* gene of the spirochete, which was used as a negative control to determine the function of the LB361 gene product in host cells, was also transfected into J774A.1 or THP-1 cells and then identified by Western Blot assay. The rabbit anti-ChpI-IgG was provided by our laboratory. In the assay, normal J774A.1 and THP-1 cells without transfection were used as the controls.

**Generation and identification of P_2_X_7_-depleted macrophages**

To determine the role of P_2_X_7_ calcium channel in extracellular Ca^2+^ influx, the P_2_X_7_ gene in J774A.1 or THP-1 cells was depleted with siRNA interference [6]. The control siRNAs (Catalog No.: D-001206-13) and mouse P_2_X_7_-siRNAs (Catalog No.: M-048023-01) or human P_2_X_7_-siRNAs (Catalog No.: M-003728-01) in siGENOME SMARTpool database and a siRNA Transfection Kit were provided by Thermo Scientific Co. (USA). Briefly, J774A.1 or THP-1 cells (10^5^ per well) were seeded in 6-well culture plates (Corning, USA) for incubation at 37°C. When the cells were 60% confluent, 25 nM P_2_X_7_-siRNAs were transfected into the cells for P_2_X_7_ gene silencing according to the manufacturer’s protocol. The two P_2_X_7_-depleted macrophages were lysed with 0.05% NaTDC-PBS [2], followed by a 500×g centrifugation at 4°C for 15 min to harvest supernatants. Using 1:500 diluted rabbit anti-human P_2_X_7_-IgG (Santa Cruz, USA) as the primary antibody and 1:3000 diluted HRP-conjugated goat anti-rabbit-IgG (Jackson ImmunoResearch) as the secondary antibody, Western Blot assay was performed to confirm the depletion of P_2_X_7_ in the two macrophages. In the assay, normal J774A.1 and THP-1 cells without P_2_X_7_ depletion were used as the controls.

**Results**

**Distribution of LA0543, LA2250 and LB361 genes in different leptospiral strains**

All the seven tested pathogenic *L. interrogans* strains but not the two non-pathogenic *L. interrogans* strains belonging to different serogroups and serovars possess LA0543 gene with 99.10%**-**100% nucleotide and 98.9%**-**100% amino acid sequence identities, and LB361 gene with 99.5%**-**100% nucleotide and 99.2%**-**100% amino acid sequence identities (Figure S2A, the sequencing data not shown). Unexpectedly, except for *L. interrogans* strain Lai, all the other eight leptospiral strains were undetectable for LA2250 gene. In particular, the three different genes amplified from *L. interrogans* serogroup Icterohaemorrhagiae serovar Lai strain Lai had 100% sequence identities compared to that in the report (GenBank accession No.: NC_004342).

**Expression and purification of target recombinant proteins**

Under inducement of IPTG, the three engineered *E. coli* BL21DE3 strains expressed the soluble recombinant proteins of LA0543, LA2250 and LB361 genes of *L. interrogans* strain Lai, respectively, and each of the purified recombinant proteins by Ni-NTA affinity chromatography showed a single band on gel after electrophoresis (Figure S2B).

**Characterization of ΔLB361 and CΔLB361 mutants**

The PCR and sequencing data confirmed the LB361 gene deletion in the ΔLB361 mutant and the LB361 gene complementation in the CΔLB361 mutant (Figure S3A, B, C and D). The Western Blot assay also confirmed the absence of LB361 gene-encoding protein in the ΔLB361 mutant and the existence of LB361 gene-encoding protein in the CΔLB361 mutant (Figure S4A). The two mutants could persistently grow in EMJH medium with typical morphology and motility and growth kinetics similar to wild-type *L. interrogans* strain Lai (data not shown).

**Characterization of LB361 gene-transfected or P_2_X_7_-depleted macrophages.**

The Western Blot assays demonstrated that LB361 gene-encoding protein in the LB361 gene-transfected J774A.1 or THP-1 cells as well as the ChpI protein in the *chpI* gene-transfected J774A.1 or THP-1 cells was detectable (Figure S4B and C), while P_2_X_7_ protein in the P_2_X_7_-depleted J774A.1 or THP-1 cells was absent (Figure S4D). Furthermore, the laser confocal microscopic examination further demonstrated that the LB361 gene-encoding protein was expressed in the LB361 gene-transfected J774A.1 or THP-1 cells with high efficiency (Figure S4E).

**References**

1. Zhang CL, Wang H, Yan J (2011) Leptospirosis prevalence in Chinese populations in the last two decades. Microbes Infect 14: 317-323.
2. Zhang L, Zhang CL, Ojcius DM, Sun D, Zhao JF, et al. (2012) The mammalian cell entry (Mce) protein of pathogenic *Leptospira* species is responsible for RGD motif-dependent infection of cells and animals. Mol Microbiol 83: 1006-1023.
3. Bauby H, Saint GI, Picardeau M (2003) Construction and complementation of the first auxotrophic mutant in the spirochaete *Leptospira meyeri*. Microbiology 149: 689-693.
4. Luo DJ, Xue F, Ojcius DM, Zhao JF, Mao YF, et al. (2010) Protein typing of major outer membrane lipoproteins from Chinese pathogenic *Leptospira* spp. and characterization of their immunogenicity. Vaccine 28: 243-255.
5. Schnoor M, Buers I, Sietmann A, Brodde MF, Hofnagel O, et al. (2009) Efﬁcient non-viral transfection of THP-1 cells. J Immunol Methods 344: 109-115.
6. Zhou D, Chen ML, Zhang YQ, Zhao ZQ (2010) Involvement of spinal microglial P2X7 receptor in generation of tolerance to morphine analgesia in rats. J Neurosci 30:8042-8047.

the primers used in this study

|  |  |  |  |
| --- | --- | --- | --- |
|  |  |  |  |
|  |  |  |  |
|  |  |  |  |
|  |  |  |  |
|  |  |  |  |
|  |  |  |  |
|  |  |  |  |
|  |  |  |  |
|  |  |  |  |
|  |  |  |  |
|  |  |  |  |
|  |  |  |  |
|  |  |  |  |
|  |  |  |  |
|  |  |  |  |
|  |  |  |  |
|  |  |  |  |
|  |  |  |  |
|  |  |  |  |
|  |  |  |  |
|  |  |  |  |
|  |  |  |  |
|  |  |  |  |
|  |  |  |  |
|  |  |  |  |
|  |  |  |  |
|  |  |  |  |
|  |  |  |  |
|  |  |  |  |
|  |  |  |  |
|  |  |  |  |
|  |  |  |  |
|  |  |  |  |
|  |  |  |  |

F: forward primer. R: reverse primer. The underlined areas indicate th
